# Response mechanisms of *Polygonatum kingianum* to temperature stress: implications for cultivation practices

**DOI:** 10.3389/fpls.2025.1645666

**Published:** 2025-11-26

**Authors:** Huali Qian, Hong Tao, Zhe Xu, Xiaolei Chen, Lei Zhang, Jiahong Dong, Pengzhang Ji

**Affiliations:** 1 School of Chinese Materia Medica and Yunnan Key Laboratory of Sustainable Utilization of Southern Medicine, Yunnan University of Chinese Medicine, Kunming, China; 2 Institute of Medicinal Plant Cultivation, Academy of Southern Medicine, Yunnan University of Chinese Medicine, Kunming, China

**Keywords:** *P. kingianum*, temperature stress, transcriptome analysis, antioxidant system, cultivation practices

## Abstract

*Polygonatum kingianum*, a medicinally significant herb indigenous to Yunnan, China, exhibits pronounced sensitivity to temperature affecting its bioactive compound accumulation and growth. However, the molecular mechanisms underlying its response to temperature stress remain poorly characterized. To elucidate these mechanisms, we integrated physiological assessments with RNA-seq analysis of *P. kingianum* under controlled temperatures (10°C, 25°C, 30°C, and 35°C). The results indicate that *P. kingianum* exhibits optimal root growth at 10–25°C but shows poor adaptation to high temperature. Temperature stress led to inhibited root growth, accompanied by decreased chlorophyll content and leaf yellowing. This stress manifested as increased levels of malondialdehyde (MDA) and hydrogen peroxide (H_2_O_2_) in the leaves, along with elevated proline accumulation. In response to oxidative damage, the activities of superoxide dismutase (SOD), peroxidase (POD), and catalase (CAT), as well as the levels of non-enzymatic antioxidants (ascorbate/ASA and glutathione/GSH), were significantly upregulated. Transcriptome analysis further revealed that these physiological changes are regulated by multiple stress-related pathways, mainly including phenylpropanoid biosynthesis, glycolysis/gluconeogenesis, starch and sucrose metabolism, and plant hormone signaling. Additionally, 19 key genes encoding reactive oxygen species scavenging enzymes were identified and functionally characterized as core regulators of thermotolerance. This study deciphers the physiological and molecular mechanisms of the temperature stress response in *P. kingianum*.

## 1 Introduction

*Polygonatum kingianum* Coll. et Hemsl. is a source material for *Polygonati Rhizoma*, and its tubers contain a variety of bioactive components with diverse pharmacological effects. Recognized as a specialty authentic medicinal herb of Yunnan Province in 2024, the increasing demand for this medicinally and culinarily valuable plant has led to a market predominantly supplied by cultivated sources (Guo et al., 2023). Predominantly distributed in Southwest China (Yunnan, Sichuan, and Guizhou), its main planting areas in Yunnan include Puer, Qujing, Yuxi, Baoshan, Lijiang, Dali, and Nujiang. During winter, Lijiang’s average temperature approximates 10 degrees Celsius, while the short-term lows can be near -5 degrees Celsius, while in summer, the average temperature in Xishuangbanna and Yuanjiang County is close to 25 degrees Celsius, with occasional short-term highs approaching 35 degrees Celsius. Climate change exacerbates extreme temperature phenomena and adversely affects plant growth, development, and physiological processes (Agüer and Haba, 2021). High temperature stress above 30°C accelerated grain filling process, decreased 1000 grain weight and yield, inhibited starch synthesis in endosperm amyloid, and finally affected yield and quality. Low temperature stress of about 3°C will reduce chlorophyll content, affect many physiological and biochemical processes of plants, and then lead to the decline of quality and yield (Mahdavi et al., 2022; Kang et al., 2023). With the growing area of *P. kingianum* growing from Xishuangbanna to Lijiang, temperature change has become a key limiting factor affecting its growth and sustainability.

Temperature significantly influences plant physiology, signaling pathways, and defense responses, which is reflected by changes in secondary metabolites (Yang et al., 2018; Bianchetti et al., 2020). These changes subsequently impact photosynthesis and stress tolerance. Photosynthesis, being temperature-sensitive (Mathur et al., 2014), is particularly vulnerable to temperature stress, which can disrupt electron transport, reduce enzyme activity, and impair carbon fixation efficiency. Photosynthetically derived reactive oxygen species (ROS) play a dual role in plant growth regulation and environmental stress responses. While acting as deleterious factors in oxidative metabolism, causing cellular damage and programmed cell death, ROS also function as essential signaling molecules for plant tolerance to biotic and abiotic stresses (Suzuki et al., 2011; Dietz et al., 2016). Temperature stress disrupts ROS homeostasis, activating plant defense mechanisms. Counteracting ROS involves non-enzymatic antioxidants like glutathione (GSH), ascorbate (ASA), and phenolics. ASA acts both as an electron donor to directly reduce ROS (Noctor and Foyer, 1998) and as a substrate in enzymatic cycles (Mittler, 2002). Additionally, plants deploy enzymatic ROS scavenging systems, including superoxide dismutase (SOD), catalase (CAT), peroxidase (POD), and proline-associated enzymes, to mitigate oxidative stress under abiotic stress conditions (Müller and Munné-Bosch, 2015; Naing et al., 2018, 2021).

Abiotic factors (such as light, moisture, temperature, and heavy metals) regulate plant growth, yield, and synthesis of secondary metabolites through complex biological mechanisms involving multi-level signal transduction and differential gene expression (Zhang et al., 2025). Cold stress can cause tissue damage or even death in different degrees and significantly affect reproductive growth at the critical stage of plant growth and development, among which male organs are usually more sensitive (Yu et al., 2025).The cold response mechanism relies on multiple signaling pathways including the CBF/DREB1 transcription factor pathway, which regulates about 10 - 20% of cold response genes (such as COR gene), significantly affecting plant cold resistance (Zhou et al., 2023). High temperature stress disrupts plant cellular homeostasis and photosynthesis, thereby inhibiting growth and altering carbon and nitrogen metabolism. In response to this stress, plants initiate multiple adaptive mechanisms that include osmotic regulation, antioxidant defense, and the induction of heat shock proteins to enhance their thermotolerance (Dubey et al., 2022; Luo et al., 2023). Under drought stress conditions, the study of *P. kingianum* showed that leaf area, relative water content, chlorophyll content and fresh weight of shoot decreased significantly, electrolyte leakage increased with the severity of stress, indicating that cell membrane stability decreased (Qian et al., 2021). In addition, combined drought and high temperature stress can lead to an increase in respiratory rate, highlighting the complexity of multiple stress interactions (Kuppusamy et al., 2023). In recent years, the effects of combined abiotic stress on plants have received increasing attention. Studies have shown that plants tend to exhibit reduced carbohydrate synthesis and increased carbohydrate consumption under complex stress conditions, ultimately resulting in stunted crop growth and decreased grain yield (Kamatchi et al., 2024). It is worth noting that some stress combinations may produce nonadditive effects. For example, moderate salt stress can significantly enhance the tolerance of *Seuvium portulacastrum* to low temperature (5 °C), indicating that there is antagonism between salinity and low temperature (Liu et al., 2025). In conclusion, plants survive by integrating signal transduction, gene expression regulation, and physiological and biochemical responses to single or combined abiotic stresses, but their response strategies and resistance performance are significantly affected by stress types, intensity, and interactions.

However, despite extensive studies on plant stress response, the molecular mechanism of *P. kingianum* response to temperature stress, especially the coordinated regulatory pathways, is still unclear. To this end, the objectives of this study were to comprehensively assess the physiological and biochemical responses of *P. kingianum* to a range of temperatures (10 °C, 25 °C, 30 °C, and 35 °C); identify key genes and pathways involved in temperature stress adaptation using multi-tissue (root, stem, leaf) transcriptome sequencing; and validate candidate genes that mediate antioxidant defense and hormone signaling. By combining physiological data with molecular mechanisms, this study aims to elucidate the complex temperature stress response network of *P. kingianum* and provide a theoretical basis for the cultivation of stress-resistant varieties and cultivation practices.

## 2 Materials and methods

### 2.1 Plant cultivation and experimental design

The material used in this experiment was 2-year-old *P. kingianum* Coll. et Hemsl., the variety identified by Pengzhang Ji, which was collected from the planting base of Sheng Yuan Tang Co., Ltd., Zhen yuan County, Puer, Yunnan Province, China (24°0′26.3″N, 101°6′37.3″E) in July 2021. To ensure initial uniformity across all treatment groups, only Select healthy, pest-free plants that visually consistent plant height, leaf width and rhizome size and then randomly assigned to the four temperature treatment groups. Based on the temperature change range of the main production areas and species distribution model of *P. kingianum* (Guo et al., 2023; Tokić et al., 2023), this study set up four temperature gradients: low temperature (LT, 10°C), normal growth temperature (CT, 25°C), moderate temperature (MT, 30°C) and high temperature (HT, 35°C) under a light/dark period of 12 h and a light intensity of 5000 lx. The experiment consisted of four treatment groups, each with three biological replicates of 20 plants each. Each plant was transplanted into a plastic pot (top diameter: 25.5 cm; bottom diameter: 16.5 cm; height: 24.0 cm) and cultured individually in a constant temperature incubator. The growth medium is a homogeneous mixture of nutrient soil, vermiculite, and humus Soil (volume ratio 1:1:1). Fertilization was not applied throughout the experiment to ensure that all observed physiological and molecular responses were clearly attributable to temperature treatment and not disturbed by nutrient supply. The plants were watered every week after 5–6:00 pm, with a fixed amount of 500 mL/pot; weeds were removed every week, and symptoms of disease were observed. Leaf sampling was conducted daily between 6:00 and 7:00 a.m. at 1, 3, 5, 7, 14, 21, 28, and 35 days of treatment. For each replicate, 5–6 leaves were randomly collected. The following physiological indices were measured: ROS levels, MDA content, superoxide anion (O^2-^) production rate, H_2_O_2_ content, and the activities of antioxidant enzymes including CAT, POD, and SOD. Additionally, we determined the contents of proline (Pro), ASA, GSH, and chlorophyll, as well as relative electrolyte leakage. At the end of the stress treatment, the number and length of adventitious roots were determined, and several parts of plants from each group were selected and placed outdoors for 1 month for the rewarming treatment. After 35 days of stress treatment and 30 days of recovery period, the rhizomes, stems and leaves of *P. kingianum* were collected from the next day of recovery, which were harvested and then washed with deionized water, after which the different tissues were stored at -80 °C, in preparation for subsequent transcriptome sequencing. Experimental research and field studies on all plant samples, including the collection of plant materials, complied with relevant institutional, national, and international guidelines and legislation. The samples were deposited in the ZWBB Herbarium, College of Chinese Medicine, Yunnan University of Chinese Medicine, Yunnan Province, with the following registration codes: ZWBB-153, ZWBB-154, ZWBB-155, and ZWBB-156.

### 2.2 Determination of plant growth and physiological indicators

After 35 days of temperature stress, the root length of *P. kingianum* was measured with a scale. The phenotype of *P. kingianum* during stress was recorded by camera. The production rate of O^2-^ was determined by NH20H method at 510 nm (Yang et al., 2020). MDA content is measured by the thiobarbituric acid-reactive-substances (TBARS), with absorption readings at 532 and 600 nm, and calculated using an extinction coefficient of 155 mm^-1^cm^-1^ (Tommasino et al., 2012). ASA content was determined by direct iodometric titration using 0.1N iodine solution (Suntornsuk et al., 2002). GSH content was measured enzymatically at 412 nm using DTNB and glutathione reductase (Rahman et al., 2006). Relative electrolyte leakage was assessed using a conductometric method. Leaf discs were immersed in deionized water, and initial conductivity (S1) was measured after incubation at 30 °C. Samples were then boiled, cooled, and final conductivity (S2) was recorded. Leakage was calculated as (S1 − S0)/(S2 − S0) × 100%, where S0 represents the conductivity of deionized water alone (Santos et al., 2019). Proline content was quantified using a commercial kit (BC0295, Beijing Solarbio Science Technology Co., Ltd., China) at 520 nm. H_2_O_2_ content was quantified using a commercial assay kit (BC3595) (Beijing Solarbio Science Technology Co., Ltd., China) by measuring absorption at 415 nm. The activity of major antioxidant enzymes was determined according to the kit (Beijing Solarbio Science Technology Co., Ltd., China). Superoxide dismutase (SOD, BC0175) activity was measured according to NBT inhibition at a wavelength of 560 nm. Peroxidase (POD, BC0095) activity was evaluated using guaiacol oxidation (ϵ = 26.6 mm^-1^ cm^-1^) at a wavelength of 470 nm. The activity of catalase (CAT, BC0205) was determined by monitoring the decomposition of H_2_O_2_ at a wavelength of 240 nm (ϵ = 39.4 mm^-1^ cm^-1^). All enzyme activities were expressed as Ug^-1^FW.The chlorophyll content was determined according to the methods of Huang et al. (2013) with slight modifications. The fresh leaves were weighed and cleaned. Leaves were then soaked in absolute ethanol, which was used as an extractant, and placed in the dark at room temperature until they became colorless or white. Anhydrous ethanol solution was used as a blank control to determine the absorbance of the extract at wavelengths of 663 nm and 645 nm by spectrophotometry.

### 2.3 Transcriptome sequencing

Three replicates of tuber, stem, and leaf samples from plants subjected to different treatments were collected, washed with deionized water, and ground in liquid nitrogen. RNA was analyzed for purity, concentration, and integrity to check RNA quality, and cDNA libraries were subsequently constructed. Raw sequencing reads underwent quality control using Trimmomatic to remove adapter sequences and filter out low-quality bases (Q<20) and N bases, yielding high-quality clean reads. *De novo* transcriptome assembly was then performed with Trinity (v2.4) to generate Unigene sequences. Read alignment to Unigenes was executed via bowtie2, followed by expression quantification (FPKM and count values) using express software. Transcriptome sequencing was performed on the Illumina platform to identify the DEGs in different tissues of *P. kingianum* from the treatment groups under different temperature conditions using the DESeq2 method based on the negative binomial distribution. A false discovery rate (FDR) < 0.05 and a |log_2_ FC| > 1 were set as the thresholds for identifying differentially expressed genes (DEGs). DEGs were identified in tubers, stems, and leaves through separate comparisons of the LT, MT, and HT groups with the CT group.

### 2.4 RNA extraction and quantitative real-time PCR

The total RNA of the tubers, stems, and leaves of *P. kingianum* was extracted by the TRIzol method, and a high-quality RNA sample was used as a template for reverse transcription. A reverse transcription kit (TSINGKE TSK301S Goldenstar™ RT6 cDNA Synthesis Kit, Tsingke Biotechnology Co., Ltd., Kunming, China) was used to generate first- strand cDNA. The primer screening was performed using TB Green^®^ Premix Ex Taq™ II (Tli RNaseH Plus) (Takara Biomedical Technology (Beijing) Co., Ltd., Kunming, China). The amplification of DNA fragments by qRT–PCR was performed using a set of gene-specific primers (**Supplementary Table S1**) and the PrimeScript™ RT reagent Kit with gDNA Eraser, a kit for real-time RT–PCR (Takara Biomedical Technology (Beijing) Co., Ltd., Kunming, China). The vendor user guide provided the main steps implemented to calculate the relative expression levels of specific genes using the 2-ΔΔCt method (Livak and Schmittgen, 2001). Three biological replicates per treatment were used to estimate the mean and standard deviation.

### 2.5 Data analysis

Data are presented as mean ± SD (n = 3). Statistical analysis was performed with IBM SPSS Statistics (Version 21.0), using one-way ANOVA to assess differences among temperature groups, followed by Tukey’s HSD *post hoc* test for significant overall effects (*P* < 0.05). Figures were generated using GraphPad Prism (Version 8.0.2) and Origin (Version 2024).

## 3 Results

### 3.1 Phenotypic changes in *P. kingianum* exposed to temperature stress

**Figure 1:**
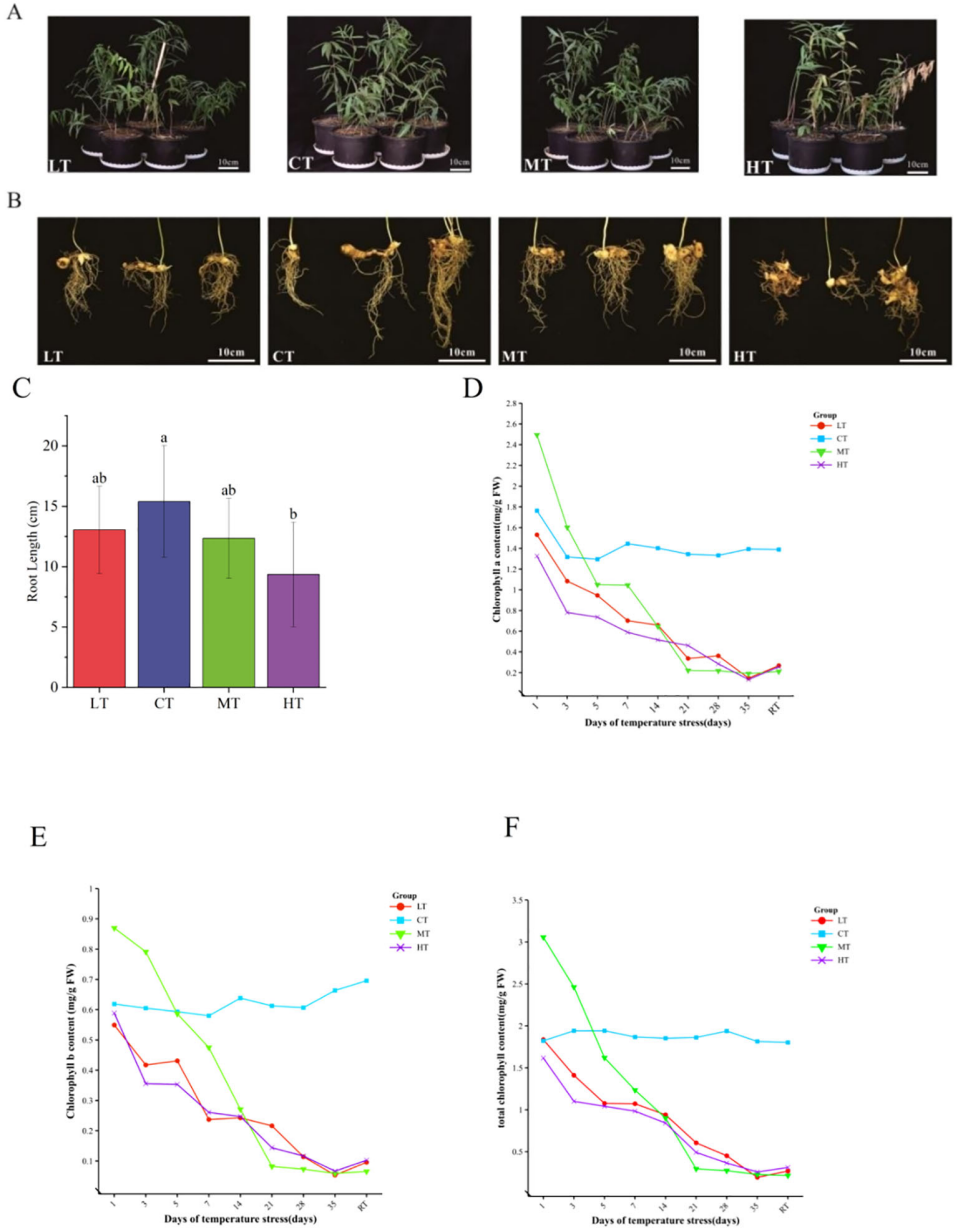
Growth of 2-year-old *P. kingianum* under different temperature treatments, namely, low temperature (LT, 10°C), control normal growth (CT, 25 °C), moderate temperature (MT, 30°C), and high temperature (HT, 35°C), for 35 days; **(A)** Growth of *P. kingianum* under temperature stress for 35 days; **(B)** Root growth of *P. kingianum* under temperature stress for 35 days; **(C)** Root length of *P. kingianum* under temperature stress for 35 days; **(D–F)** Changes in the contents of chlorophyll a, chlorophyll b, and total chlorophyll after the exposure of plants to temperature stress for 1 ~ 35 days and return to normal growing temperatures for 30 days (RT, 25°C). Data are means ± SD (n=3). Different lowercase letters above bars indicate significant differences (*P* < 0.05) according to one-way ANOVA with Tukey’s HSD test.

Compared with plants grown at normal CT treatments, *P. kingianum* roots subjected to different temperature stresses were shortened and the number of segments decreased (**Figure 1B**). At 35°C, root length decreased significantly by 39.2% (*P* < 0.05); at 10°C and 30°C, root length decreased by 15.23% and 19.78%, respectively (**Figure 1C**). In addition, leaf yellowing and scorch were more severe in the high temperature group than in the other groups (**Figure 1A**), while the low temperature group caused less damage to leaves. Although low LT, MT and HT treatments all led to significant decreases in the contents of chlorophyll a, chlorophyll b, and total chlorophyll, the most pronounced decrease was observed under low-temperature conditions (**Figures 1D–F**). The chlorophyll content in the control group remained stable at all time points. Therefore, when the plants were restored to optimal growth conditions, the chlorophyll content showed a significant upward trend.

### 3.2 Physiological and biochemical responses of leaves of *P. kingianum* to different temperature stress levels

**Figure 2:**
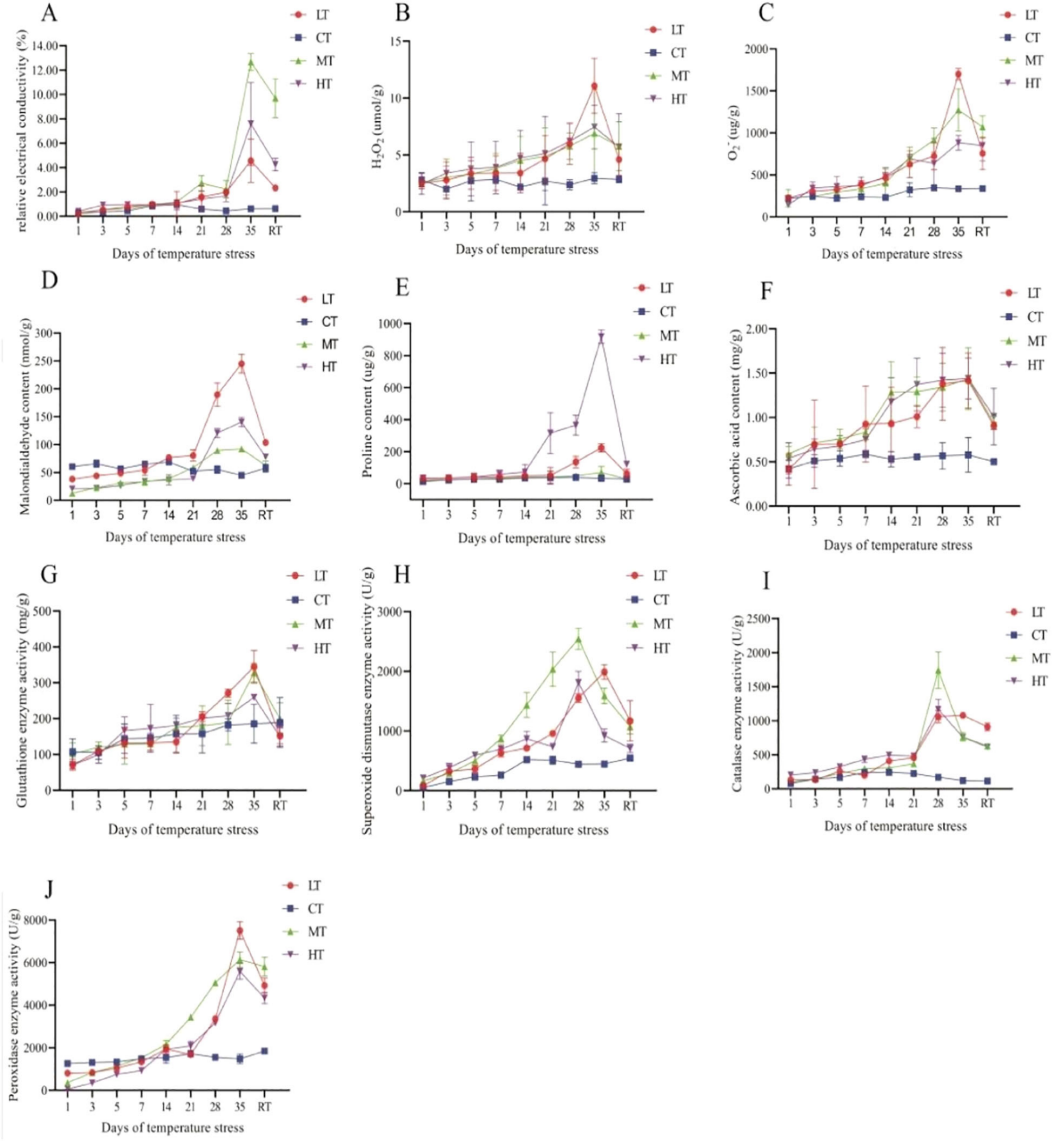
Dynamic physiological responses of *P. kingianum* to a 35-day temperature stress and 30-day recovery period. **(A)** Relative electrical conductivity; **(B)** H_2_O_2_ content; **(C)** superoxide anion (O^2-^) content; **(D)** malondialdehyde content; **(E)** Proline content; **(F)** Ascorbic acid content; **(G)** Glutathione enzyme activity; **(H)** Superoxide dismutase activity; **(I)** Catalase enzyme activity; **(J)** Peroxidase enzyme activity. Data are presented as mean ± SD (n = 3). LT, 10 °C; CT, 25 °C; MT, 30 °C; HT, 35 °C; RT, ambient conditions.

Under optimal temperature conditions, the plasma membrane of *P. kingianum* leaves maintains homeostasis, with a relative electrolyte leakage of only 0.61%. However, after 35 days of temperature stress treatment, the relative electrolyte leakage in the LT, MT, HT, groups increased significantly to 4.56%, 12.68%, and 7.59%, respectively, which were 7, 20, and 12 times higher than that of the CT. These results indicate that temperature stress caused severe damage to the plasma membrane integrity, with the medium-temperature (30 °C) treatment causing the most significant damage to the cell membrane (**Figure 2A**).

As shown in **Figures 2C, D**, the physiological indices of O^2-^ production rate and H_2_O_2_ and MDA contents in *P. kingianum* leaves under temperature stress were determined. At the initial stage of stress, these indices showed no significant changes. However, after 14 days of stress treatment, the O^2-^ production rate and H_2_O_2_ content increased significantly. After 21 days of stress treatment, the MDA content also increased significantly; at 35 days, the MDA content in the LT, MT, and HT groups increased sharply, showing an increase of approximately 425%, 99%, and 200% compared to that in the control group, respectively, indicating that serious oxidative damage occurred under long-term temperature stress. Notably, the O^2-^ production rate and H_2_O_2_ and MDA contents in LT-, MT-, and HT-treated plants returned to levels close to those of CT plants after a 30-day recovery period.

Additionally, proline (Pro) content remained stable during the initial growth phase but increased significantly after 14 days of stress treatment. The increase was more pronounced under HT stress compared to LT stress, although both conditions induced a rise in Pro accumulation. Under temperature stress, significant changes were also observed in the physiological indices of ASA, GSH, SOD, CAT, and POD In *P. kingianum* leaves. The ASA content increased with prolonged stress duration, and this change became more pronounced after 14 days. Similarly, the trends in GSH content and POD activity were consistent, both gradually increasing with prolonged stress duration. At 35 days, the contents of non-enzymatic antioxidants (ASA and GSH) in the LT treatment were significantly higher than those in the CT treatment. The GSH content had increased by 86%, and the ASA content had increased by 145%. Meanwhile, the activities of SOD, CAT, and POD in all treatment groups (LT, MT, HT) exceeded those of the control group. The activities of SOD and CAT initially increased, peaked at 28 days of stress treatment, and then decreased. After a 30-day recovery period, the activities of ASA, GSH, SOD, CAT, and POD decreased to levels close to those of the control group (**Figures 2F–J**).

### 3.3 Transcriptome responses of *P. kingianum* to temperature stress

#### 3.3.1 RNA-seq data quality and differentially expressed genes

A total of 246.5 Gb of clean data were obtained by transcriptome sequencing of the tuber, stem, and leaf of *P. kingianum*, and the effective volume of distribution of the data from each sample was 6.39 ~ 7.25 Gb; the distribution of Q30 bases was 92.62 ~ 94.06%, and the average GC content was 48.8%. A total of 94428 unigenes strips with a total length of 87551651 bp and an average length of 927.18 bp were spliced. The database annotation results for the unigenes were as follows: 51414 (54.45%) genes from the sequenced library were annotated to the NR database; 37117 (39.31%) genes were annotated to the SWISS-PROT database; 12614 (13.36%) genes were annotated to the KEGG database; 29541 (31.28%) genes were annotated to the KOG library; 46102 (48.82%) genes were annotated to the eggNOG database; 32703 (34.63%) genes were annotated to the GO database; and 28060 (29.72%) genes were annotated to the Pfam database. After that, the reads were compared to the unigenes, and the alignment rate was 89.84 ~ 92.48%. These results indicate that the sequencing data obtained from the transcriptome analysis were of high quality and met the requirements for performing subsequent bioinformatics analysis.

**Figure 3:**
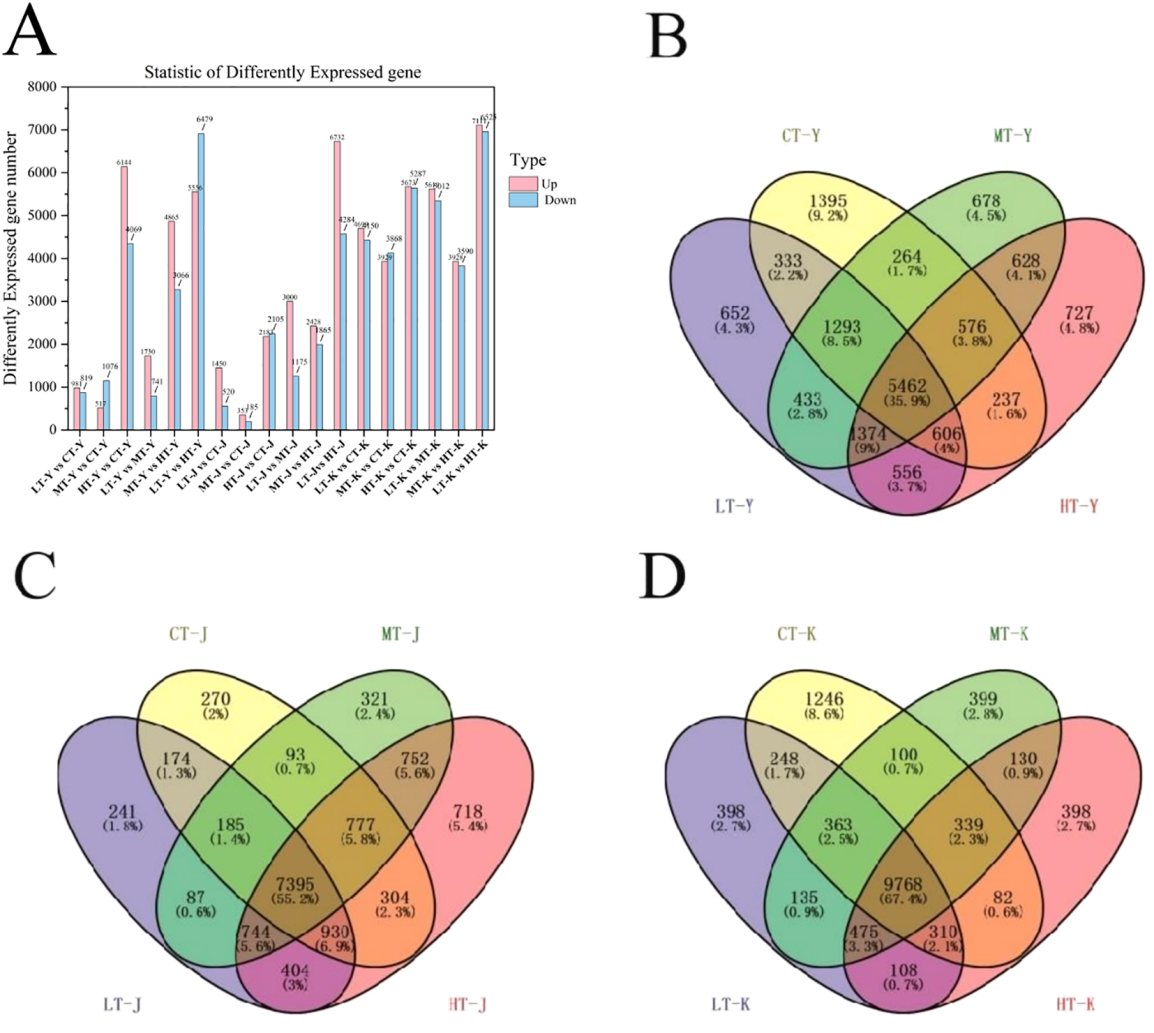
Analysis of differentially expressed genes (DEGs) in P. kingianum under temperature stress. **(A)** Number of upregulated and downregulated DEGs in pairwise comparisons between temperature treatments. **(B–D)** Venn diagrams showing unique and shared DEGs across four temperature treatments in **(B)** leaves, **(C)** stems, and **(D)** tubers. DEGs were filtered with FDR< 0.05 and |log_2_ FC| > 1. Y, leaf; J, stem; K, tuber; LT, 10°C; CT, 25°C; MT, 30°C; HT, 35°C.

In this study, differentially expressed genes (DEGs) were identified using the thresholds of |log_2_ FC| > 1 and FDR< 0.05.1970, 538, 4288, 4175, 4293, and 11014 DEGs; were found in the stem between LT-J vs. CT-J, MT-J vs. CT-J, HT-J vs. CT-J, MT-J vs. LT-J, HT-J vs. MT-J and LT-J vs. HT-J, respectively. 8849, 7797, 10960, 10631, 7518 and 13636 DEGs were found among tubers LT-K vs. CT-K, MT-K vs. CT-K, HT-K vs. CT-K, MT-K vs. LT-K, HT-K vs. MT-K and LT-K vs. HT-K, respectively. Among the leaves, 1800, 1258, 10213, 2471, 7931 and 12035 DEGs were found between LT-Y vs. CT-Y, MT-Y vs. CT-Y, HT-Y vs. CT-Y, MT-Y vs. LT-Y, HT-Y vs. MT-Y and LT-Y vs. HT-Y respectively (**Figure 3A**). In addition, in the leaves, 5462 DEGs were common among LT-Y, CT-Y, MT-Y, and HT-Y (**Figure 3B**). Among the stems, 7395 DEGs were shared among LT-J, CT-J, MT-J and HT-J (**Figure 3C**), and among the tubers, 9768 DEGs were shared among LT-K, CT-K, MT-K, and HT-K (**Figure 3D**).

#### 3.3.2 Gene ontology and Kyoto encyclopedia of genes and genomes pathway enrichment analyses of DEGs

**Figure 4:**
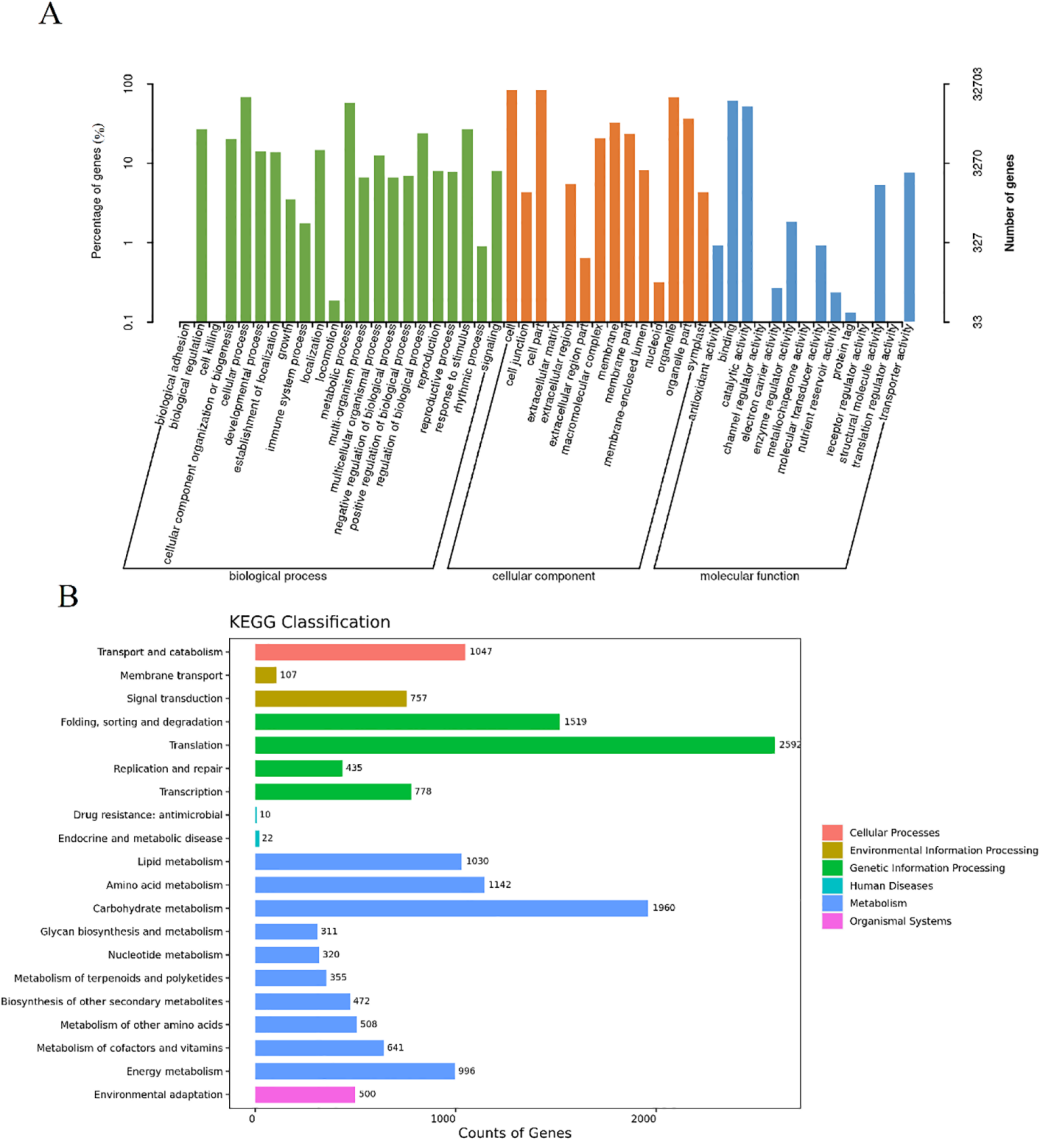
Functional classification of unigenes. **(A)** Gene Ontology (GO) classification. **(B)** Kyoto Encyclopedia of Genes and Genomes (KEGG) pathway enrichment classification. Unigenes were aligned to the GO and KEGG databases using Diamond (e-value < 1e-5). GO terms were assigned based on Swiss-Prot homologs, and KEGG pathway enrichment analysis was performed using KOBAS.

GO enrichment analysis revealed that a total of 1104 DEGs in the three comparison groups, LT-Y vs. CT-Y, MT-Y vs. CT-Y, and HT-Y vs. CT-Y, were annotated to this database. Among them, 77 DEGs were involved in biological processes, especially cellular and metabolic processes; 121 DEGs were enriched in cellular components, especially cells, cellular fractions, and organelles; and 220 DEGs were involved in molecular functions, especially binding and catalytic activity. In the stem, a total of 494 DEGs in the 3 comparison groups, namely, LT-J vs. CT-J, MT-J vs. CT-J, and HT-J vs. CT-J, were annotated to the GO database, among which 35 DEGs were involved in biological processes, especially cellular and metabolic processes; 72 DEGs were enriched in cellular components, especially cells, cellular fractions, and organelles; and 101 DEGs were involved in molecular functions, especially binding and catalytic activity. In the tubers, however, a total of 3182 DEGs in the 3 comparison groups-LT-K vs. CT-K, MT-K vs. CT-K, and HT-K vs. CT-K were annotated to the GO database. Among them, 170 DEGs were involved in biological processes, especially cellular and metabolic processes; the cellular components enriched by 390 DEGs were cells, cell fractions, and organelles; and for molecular functions, 656 DEGs were involved mainly in binding and catalytic activity (**Figure 4A**).

KEGG pathway enrichment analysis revealed that several metabolic pathways in *P. kingianum*, including those involved in carbon metabolism, lipid metabolism, amino acid synthesis, and energy metabolism, may be involved in the temperature stress response. In the three comparison groups LT vs. CT, MT vs. CT and HT vs. CT, 176, 76 and 928 differentially expressed genes (|log_2_ FC|>1, FDR<0.05) were annotated into KEGG database in leaves, tubers and stems, respectively. Among them, most genes in the leaves were enriched in pathways such as the biogenesis of ribosomes (35 DEGs), and starch and sucrose metabolism (7 DEGs). Most genes in the stem were enriched in pathways such as linoleic acid metabolism (8 DEGs), α-linoleic acid metabolism (7 DEGs), and phenylpropane biosynthesis (5 DEGs). Most genes in the tubers were enriched in pathways such as biogenesis of ribosomes (23 DEGs), phenylpropane biosynthesis (18 DEGs), plant hormone signaling (19 DEGs), amino sugar and sugar metabolism (12 DEGs), and glycolysis (12 DEGs) (**Figure 4B**). Therefore, we used clustered heatmaps to investigate the genes and metabolic pathways that respond to temperature stress, particularly the biosynthesis of secondary metabolites, carbon metabolism, lipid metabolism, and metabolism of other amino acids.

### 3.4 Coordinately regulated stress-responsive genes in key metabolic pathways

#### 3.4.1 Genes involved in phenylpropanoid biosynthesis

**Figure 5:**
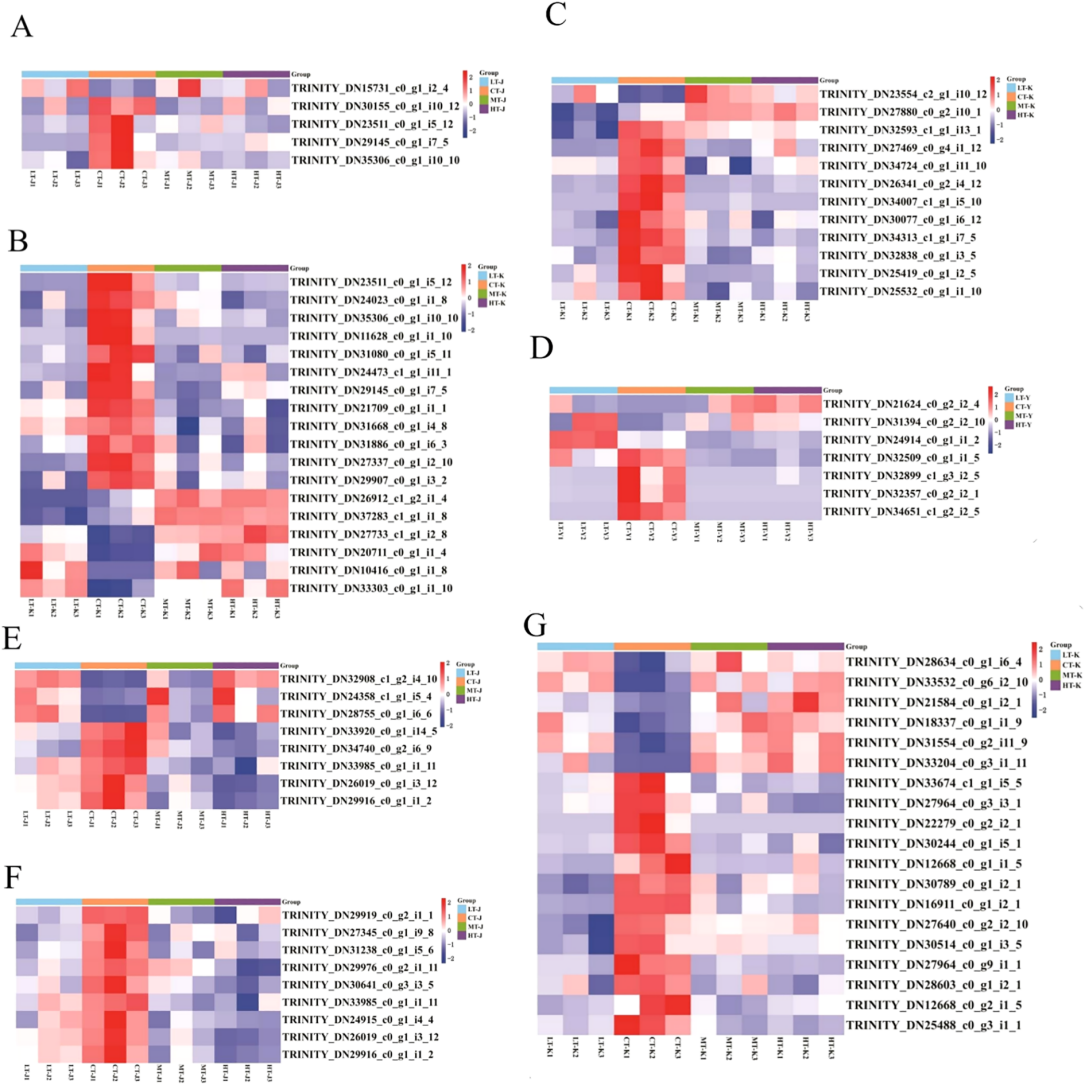
Heatmap of DEGs involved in phenylpropane biosynthesis, central carbon metabolism, linoleic acid metabolism, and plant hormone signal transduction. **(A)** Heatmap of DEGs involved in phenylpropane biosynthesis in the stem; **(B)** heatmap of DEGs involved in phenylpropane biosynthesis in the tuber. **(C)** Heatmap of genes associated with central carbon metabolism in the tuber; **(D)** Heatmap of genes associated with central carbon metabolism in the leaf. **(E)** Heatmap of genes associated with linoleic acid metabolism in the stems; **(F)** Heatmap of genes associated with α-linoleic acid metabolism in the stems. **(G)**Heatmap of differentially expressed genes related to plant hormone signal transduction. Note: Functional annotations for all genes shown in this figure are provided in **Supplementary Table S2**. Y, leaf; J, stem; K, tuber; LT, 10°C; CT, 25°C; MT, 30°C; HT, 35°C.

In this study, a total of 23 common DEGs were found to be involved in the phenylpropane biosynthesis pathway (ko00940), of which 18 DEGs were in the tuber of *P. kingianum* (**Figure 5B**). These genes encoded caffeoyl-CoA-O-methyltransferase (CCoAOMT), cinnamyl alcohol dehydrogenase (CAD) (K22395), β-glucosidase (bglB), scopoletin glucosyltransferase (TOGT1), peroxidase (POD), and 4-coumarate-CoA ligase (4CL). The genes that are significantly upregulated in *P. kingianum* following temperature stress include two genes encoding peroxidase (TRINITY-DN10416-c0-g1-i1–8 and TRINITY-DN27733-c1-g1-i2-8), one gene encoding cinnamyl alcohol dehydrogenase (TRINITY-DN20711-c0-g1-i1-4), and one gene encoding β-glucosidase (TRINITY-DN33303-c0-g1-i1-10) (**Supplementary Table S2**). Among them, 5 DEGs were involved in phenylpropane biosynthesis in the stem of *P. kingianum* (**Figure 5A**), which encoded peroxiredoxin 6 (a 1-Cysperoxiredoxin (Prdx6) that reduces peroxides), caffeoyl-CoA-O-methyltransferase (CCoAOMT), and peroxidase (POD). The Gene encoding peroxiredoxin 6 (1-Cys peroxiredoxin) (TRINITY-DN15731-c0-g1-i2-4) was upregulated in LT, CT, and MT (**Supplementary Table S2**), in response to ROS accumulation under temperature stress with the increase in antioxidant enzyme activities such as POD and SOD (**Figures 2F-J**).

#### 3.4.2 Genes involved in central carbon metabolism

**Figure 6:**
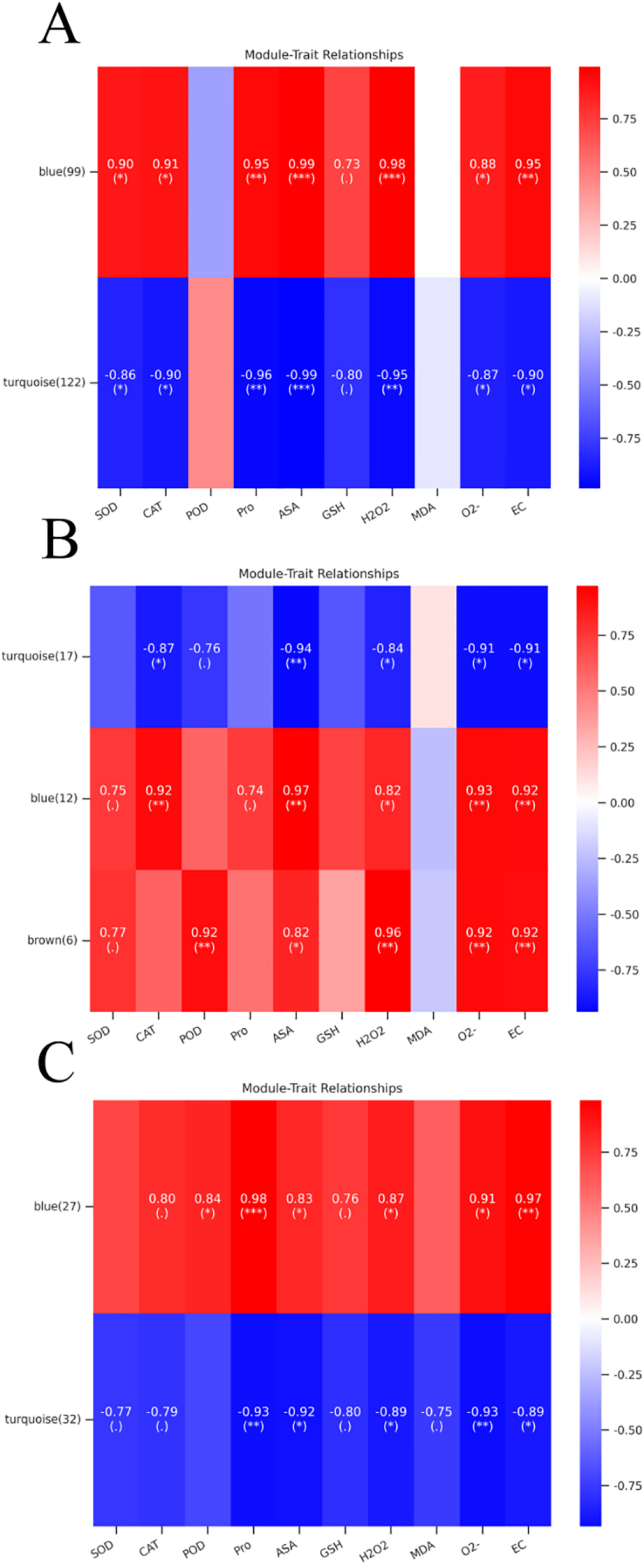
Heatmaps of module-trait relationships in leaves, constructed using weighted gene co-expression network analysis (WGCNA). **(A)** Module-trait correlations: HT vs CT. **(B)** Module-trait correlations: MT vs CT. **(C)** Module-trait correlations: LT vs CT. Modules (vertical axis); phenotypic traits (horizontal axis). Positive correlation: red; negative: green. Correlation coefficients and p-values shown in cells; significance denoted by asterisks (*P < 0.05; **P < 0.01, ***P<0.001).

A total of 19 DEGs were involved in the carbon metabolism pathway in *P. kingianum*, of which 12 were involved in the glycolysis pathway (ko00010) in the tuber of *P. kingianum* (**Figure 5C**). These genes encoded 6-phosphofructokinase 1 (PFK 1), hexokinase (HK), glucose-6-phosphate 1- epimerase (EC: 5.1.3.15), aldehyde dehydrogenase family 7 member A1 (ALDH7A1), phosphoglucomutase (PGM), alcohol dehydrogenase (NADP+) (encoded by the AKR1A1 gene), pyruvate kinase (PK), phosphoenolpyruvate carboxykinase (ATP) (EC.4.1.1.49), glyceraldehyde-3-phosphate dehydrogenase (GAPDH), and triosephosphate isomerase (TPI). In this pathway, the gene TRINITY-DN23554-c2-g1-i10–12 encoding 6-phosphofructokinase 1 (PFK 1) was significantly differentially expressed in LT, MT, and HT, indicating that temperature stress stimulated the transcription of this gene (**Figure 5D**). Among them, 7 DEGs, as shown in **Figure 6B**, encoding glycogen phosphorylase (PYG), β-amylase (EC.3.2.1.2), β- glucosidase (bglX), 4-α-glucanotransferase (malQ), isoamylase (ISA), and sucrose synthase (EC.2.4.1.13), were involved in the starch and sucrose metabolism pathway (ko00500) in *P. kingianum* leaves. In this pathway, the genes TRINITY-DN21624-c0-g2-i2–4 and TRINITY-DN31394-c0-g2-i2-10, which encode glycogen phosphorylase 4 (PYG) and β-glucosidase (bglX), respectively, were significantly differentially expressed under temperature stress (**Supplementary Table S2**).

#### 3.4.3 Lipid metabolism-related genes

Seventeen DEGs were enriched in the pathways of linoleic acid metabolism (ko00591) and α-linoleic acid metabolism (ko00592). Among them, 8 DEGs, as shown in **Figure 5E** were involved in linoleic acid metabolism and encoded the proteins linoleate 9S-lipoxygenase (LOX1_5) and lipoxygenase (LOX2S). Nine DEGs were involved in α-linoleic acid metabolism and encoded 12-oxophytodienoic acid reductase (OPR), lipoxygenase (LOX-2S), acyl-CoA oxidase (EC 1.3.3.6), and hydroperoxide dehydratase (AOS) (**Figure 5F**). In the linoleic acid metabolism pathway, three genes, including TRINITY_DN24358_c1_g1_i5_4, TRINITY_DN28755_c0_g1_i6_6, and TRINITY_DN32908_c1_g2_i4_ 10 encoding LOX1_5, were significantly upregulated and expressed in the LT, CT, and MT treatment groups.

#### 3.4.4 Genes related to plant hormone signal transduction

A total of 19 DEGs, as shown in **Figure 5G**, were identified in the tuber of *P. kingianum*. These DEGs were involved in the plant hormone signal transduction pathway (ko04075), encoding transport inhibitor response 1 (TIR1), protein phosphatase 2C (PP2C), phytochrome-interacting factor 3 (PIF3), jasmonate ZIM domain (JAZ) protein, xyloglucosyl transferase TCH4 (TCH4), and other related enzymes. Among them, the expression levels of 6 related genes encoding PR1, TGA, BIN2, BSK, and PP2C were significantly increased under temperature stress (**Supplementary Table S2**), which is speculated to be closely related to the response of *P. kingianum* to temperature stress.

### 3.5 WGCNA reveals co-expression modules related to temperature stress

Under temperature stress, significant changes in physiological and biochemical indicators were observed in *P. kingianum*. To investigate the correlation between genes and phenotypic traits, weighted gene co-expression network analysis (WGCNA) was employed to construct co-expression networks between differentially expressed genes (DEGs) selected from pathways significantly enriched after co-expression analysis of three comparisons (HT vs CT, MT vs CT, and LT vs CT) and phenotypic traits.

In the HT vs CT comparison group, an initial analysis of 232 genes across six biological samples was conducted, followed by filtering out genes with low expression variation (standard deviation ≤ 0.5), resulting in 221 retained genes. After merging highly similar small modules, two distinct modules were identified (**Figure 6A**): the turquoise module (122 genes) and the blue module (99 genes). The blue module exhibited a highly significant positive correlation with ASA (*P* < 0.001) and significant correlations with Pro and H_2_O_2_, while the turquoise module showed significant negative correlations with ASA and H_2_O_2_ (*P* < 0.001) and Pro (*P* < 0.01). Both blue and turquoise modules showed enrichment in phenylpropanoid biosynthesis (ko00940), α-linolenic acid metabolism (ko00592), and starch and sucrose metabolism (ko00500), with the blue module uniquely enriched in cyano amino acid metabolism (ko00460) (**Supplementary Figure S1B**). Within the phenylpropanoid biosynthesis pathway, the core genes of the blue modules, TRINITY_DN34221_c0_g1_i5_9, which drives phenylpropane metabolism, and TRINITY_DN16683_c0_g1_i1_9, a key gene for antioxidant defense. The core gene TRINITY_DN29907_c0_g1_i3_2 of turquoise modules, involved in phenylpropane biosynthesis, regulates phenolic glycoside synthesis. In the starch and sucrose metabolism pathway, the core gene TRINITY_DN33303_c0_g1_i1_10 of the blue module is involved in the catabolism of cellulose. The core gene TRINITY_DN31510_c0_g1_i2_9 of the turquoise module is involved in starch and sucrose metabolism (**Supplementary Table S4**).

In MT vs CT, a total of 35 genes participated in constructing WGCNA analysis, and finally 3 modules were obtained (**Figure 6B**), the genes in all three modules were significantly enriched in starch and sucrose metabolism (**Supplementary Figure S1D**). Among which the number of turquoise module genes was 17, which showed a significant negative correlation with ASA (*P* < 0.01). The 12 genes of blue module and 6 genes of brown module were positively correlated with phenotypic characteristics, among which the correlation between blue module and ASA was the highest, and brown module had the same correlation coefficient with POD, O^2-^ and EC. The core gene of the blue module is TRINITY_DN31394_c0_g2_i2_10, which encodes β-glucosidase as mentioned in result 2.4.2. The core gene TRINITY_DN32509_c0_g1_i1_5 of the turquoise module, which is involved in the starch and sucrose metabolic pathways and catalyses branched-chain starch catabolism. (**Supplementary Table S4**).

In the comparison group between LT and CT, 60 genes were categorized into two distinct modules (**Figure 6C**). Most of the genes within these modules were predominantly enriched in the phenylpropanoid biosynthesis pathway (Ko00940) and the α-linolenic acid metabolism pathway (Ko00592) (**Supplementary Figure S1F**). The core genes identified included TRINITY_DN28436_c0_g2_i2_5, TRINITY_DN34238_c0_g1_i1_2, and TRINITY_DN30857_c0_g2_i1_9(**Supplementary Table S4**). Notably, both the blue module and the turquoise module exhibited the highest correlation with pro, as well as strong correlations with O^2-^ and EC.

### 3.6 Protein–protein interaction network analysis of the common stress-responsive DEGs

PPI network analysis of co-expressed differentially expressed genes (DEGs) in *P. kingianum* tubers identified 59 key genes (**Supplementary Figure S2**), including 11 potentially involved in temperature stress responses (**Supplementary Table S5**). Expression analysis revealed downregulation of these genes in tubers under stress, contrasting with upregulation in controls. Notably, TRINITY_DN34007_c1_g1_i5_10—encoding glyceraldehyde-3-phosphate dehydrogenase linked to glycolysis, gluconeogenesis, and carbon metabolism—was significantly downregulated, suggesting disrupted osmoregulatory synthesis in central carbon metabolism. Another critical gene, encoding disease resistance protein RPS2 (involved in plant-pathogen interactions), exhibited reduced expression under stress, indicating heightened pathogen susceptibility in temperature-stressed plants. Six additional genes (e.g., TRINITY_DN11775_c0_g1_i110) were associated with replication and repair processes, further elucidating stress adaptation mechanisms.

### 3.7 Expression analysis of transcription factors in *P. kingianum* in response to different temperature stress levels

**Figure 7:**
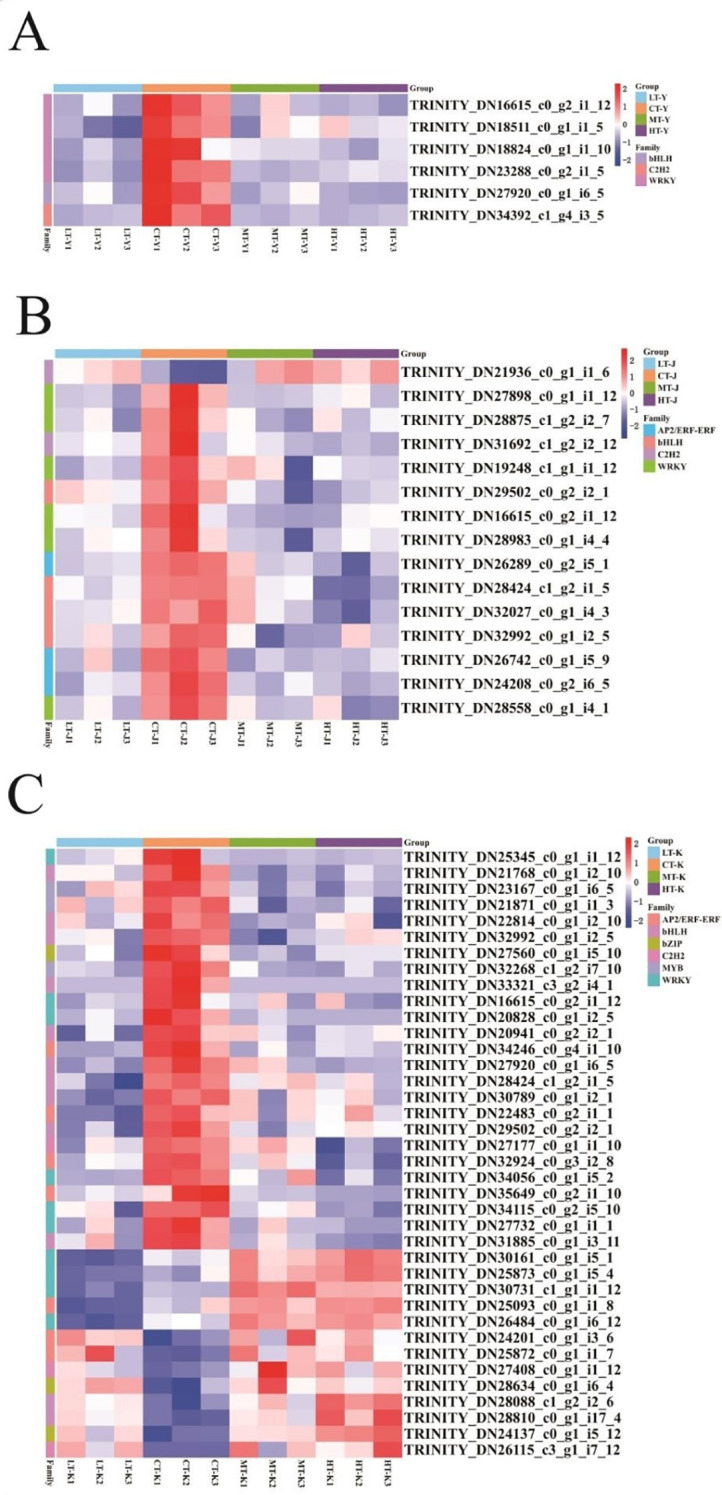
Heatmap showing relevant gene expression by the screening of key transcription factors; key genes associated with the transcription factors screened in **(A)** leaves; **(B)** stems; and **(C)** tubers. Y, leaf; J, stem; K, tuber; LT, 10°C; CT, 25°C; MT, 30°C; HT, 35°C.

The transcriptional dynamics of key transcription factor (TF) families in *P. kingianum* under temperature stress (10 °C and 35 °C) were analyzed. In tubers, three AP2/ERF genes exhibited distinct expression patterns: TRINITY_DN25093_c0_g1_i1_8 was upregulated at 35°C but downregulated at 10 °C, while TRINITY_DN24201_c0_g1_i3_6 and TRINITY_DN25872_c0_g1_i1_7 showed consistent upregulation under both temperature extremes. PPI network analysis and expression validation (**Supplementary Figure S2**, **Supplementary Table S6**) confirmed their regulatory roles. High-temperature stress induced significant upregulation of four WRKY genes (TRINITY_DN30161_c0_g1_i5_1, TRINITY_DN25873_c0_g1_i5_4, TRINITY_DN30731_c1_g1_i1_12, TRINITY_DN26484_c0_g1_i6_12) in tubers, while TRINITY_DN18824_c0_g1_i1_0 in leaves was downregulated. Two bZIP genes (TRINITY_DN24137_c0_g1_i5_12, TRINITY_DN28634_c0_g1_i6_4) in tubers displayed upregulated expression, correlating with antioxidant enzyme activity and proline accumulation (**Supplementary Table S7**, **Figure 7**), suggesting ABA-mediated regulation of CAT/APX genes. Additionally, C_2_H_2_ (TRINITY_DN21936_c0_g1_i1_6, RINITY_DN27408_c0_g1_i112, RINITY_DN26115_c3_g1_i7_12) and bHLH (TRINITY_DN28088_c1_g2_i2_6, TRINITY_DN28810_c0_g1_i17_4) TFs were consistently upregulated, aligning with AP2/ERF and bZIP stress responses. These TFs are implicated in temperature stress tolerance mechanisms.

### 3.8 Validation of qRT–PCR assay

**Figure 8:**
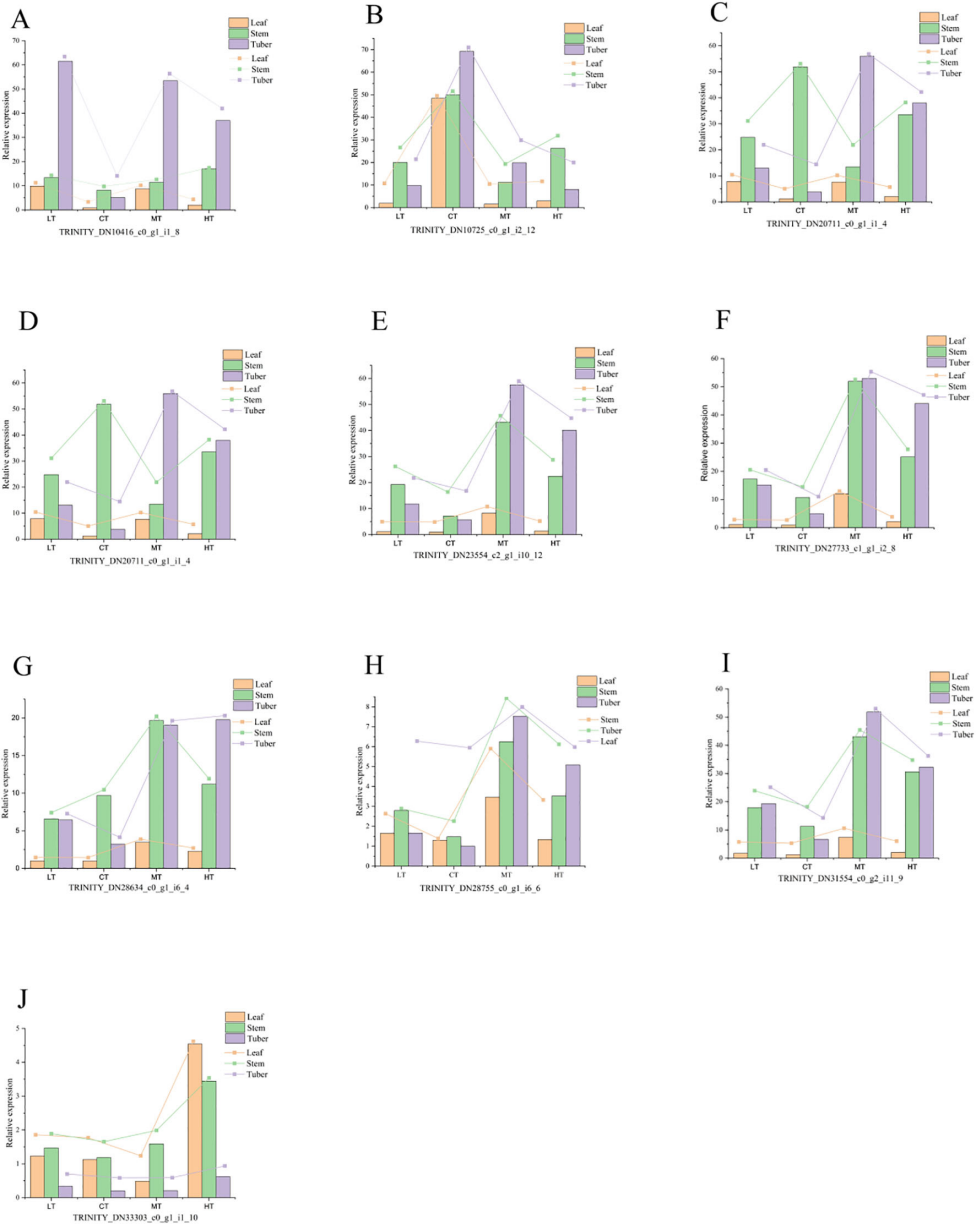
Quantitative analysis of the expression of 10 genes in different tissues of *P. kingianum* under different stress levels. Panels **(A–J)** represent the expression levels of the 10 respective genes.Note: Y, leaf; J, stem; K, tuber; LT, 10 °C; CT, 25 °C; MT, 30 °C; HT, 35 °C.

Seventeen key genes screened from the main metabolic pathways, including phenylpropanoid biosynthesis, linoleic acid metabolism, α-linoleic acid metabolism, glycolysis, and plant hormone signaling, as well as 9 DEGs, were selected for qRT–PCR verification. Consistent with transcriptomic results, 9 genes showed upregulated expression under both low- and high-temperature stress. In *P. kingianum* tubers, two peroxidase genes (TRINITY_DN10416_c0_g1_i1_8, TRINITY_DN27733_c1_g1_i2_8) in phenylpropanoid biosynthesis, along with cinnamyl alcohol dehydrogenase (TRINITY_DN20711_c0_g1_i1_4) and β-glucosidase (TRINITY_DN33303_c0_g1_i1_10), exhibited significant upregulation at 10°C, 30°C, and 35°C. The glycolysis-related 6-phosphate fructokinase 1 gene (TRINITY_DN23554_c2_g1_i10_12) and plant hormone signaling genes encoding TGA transcription factors (TRINITY_DN28634_c0_g1_i6_4) and brassinosteroid insensitive 2 proteins (TRINITY_DN31554_c0_g2_i11_9) were also upregulated in tubers under temperature treatments. In stems, peroxiredoxin 6 (TRINITY_DN15731_c0_g1_i2_4) and linoleic acid 9S-lipoxygenase (TRINITY_DN28755_c0_g1_i6_6) showed temperature-responsive upregulation (**Figure 8**). These results demonstrate coordinated activation of stress-responsive pathways across tissues under thermal challenges.

## 4 Discussion

### 4.1 Morphological and physiological response of *P. kingianum* to temperature stress

Temperature stress inhibited the growth of *P. kingianum*, especially under high temperature conditions, root length was significantly shortened (*P* < 0.05) (**Figure 1**). This is highly consistent with research findings in cotton. The results showed that high temperature changed root architecture by accelerating root meristem division and lateral root primordia development and finally caused serious inhibition of root growth at the expense of taproot elongation (Fan et al., 2022). In contrast, the inhibition of root length by low temperature stress was weaker. Under low temperature, the root structure is less damaged, and the cell metabolism and membrane integrity are easier to maintain (Han et al., 2022). High and low temperature treatments also destroy chloroplast membrane structure, inhibit chlorophyll synthesis, and accelerate its degradation. However, moderate temperature treatment could increase chlorophyll content temporarily, which could be regarded as a positive adaptive physiological response (Zhao et al., 2020; Rossi et al., 2023; Xue et al., 2023). The application of combined forest-medicine planting and shading techniques can alleviate the above-mentioned stress effects by improving the microclimate (Zhao et al., 2025).This study also further revealed the different physiological damages caused by low temperature and high temperature stress and the adaptation mechanisms of plants: After 35 days of treatment, the content of MDA under LT treatment was significantly higher than that under MT and HT treatment (**Figure 2D**), consistent with the accumulation trends of H_2_O_2_ and O^2−^, indicating that low temperature causes more severe membrane lipid peroxidation, which is in line with the typical characteristics of cold damage (Sadžak et al., 2020; Zheng et al., 2024). Moderate to high temperature treatment, however, led to more severe electrolyte extravasation (**Figure 2A**), indicating more severe damage to cell membrane integrity, which is related to membrane protein denaturation and inactivation of key enzymes in chloroplasts and mitochondria (Gul et al., 2021). In addition, proline accumulation was significantly higher under HT than LT (**Figure 2E**). High temperatures cause stomata to close to reduce water loss, resulting in impaired CO^2-^ fixation, excessive reduction of the photosynthetic electron transport chain and production of excess NADPH, while proline biosynthesis helps consume excess NADPH, thereby mitigating oxidative damage (Mulugeta et al., 2023). Therefore, in the Yuanjiang dry-hot valley and other high temperature areas where the summer temperature is often higher than 33.8 °C, cooling measures such as shading and sprinkler irrigation should be taken, and water management should be taken as the core. Nitrogen fertilizer application can promote proline accumulation, or brassinolide (e.g. EBL) can be applied externally to reduce proline degradation (Seleiman et al., 2023; Zhao et al., 2023); and in the low temperature stress area, it is important to adopt measures to Maintain the temperature and stabilize the membrane structure (Zhong et al., 2024). The activities of ROS, MDA, Pro, non-enzymatic antioxidants (ASA, GSH), and antioxidant enzymes (SOD, CAT) all increased under stress conditions (**Figure 2**), indicating that the antioxidant defense system of the plants was activated. However, under long-term low-temperature stress, the activities of key enzymes such as SOD and CAT decreased after reaching the peak on the 28th day (**Figures 2H, I**), indicating that the continuous generation of ROS and the later attenuation of antioxidant enzyme activities jointly exacerbated oxidative damage (Alharby et al., 2021; Goel et al., 2022). In conclusion, implementing targeted cultivation management based on the physiological response differences under different temperature stresses is of great significance for improving the yield and quality of *P. sibiricum*.

### 4.2 Response mechanisms of *P. kingianum* to temperature stress

**Figure 9:**
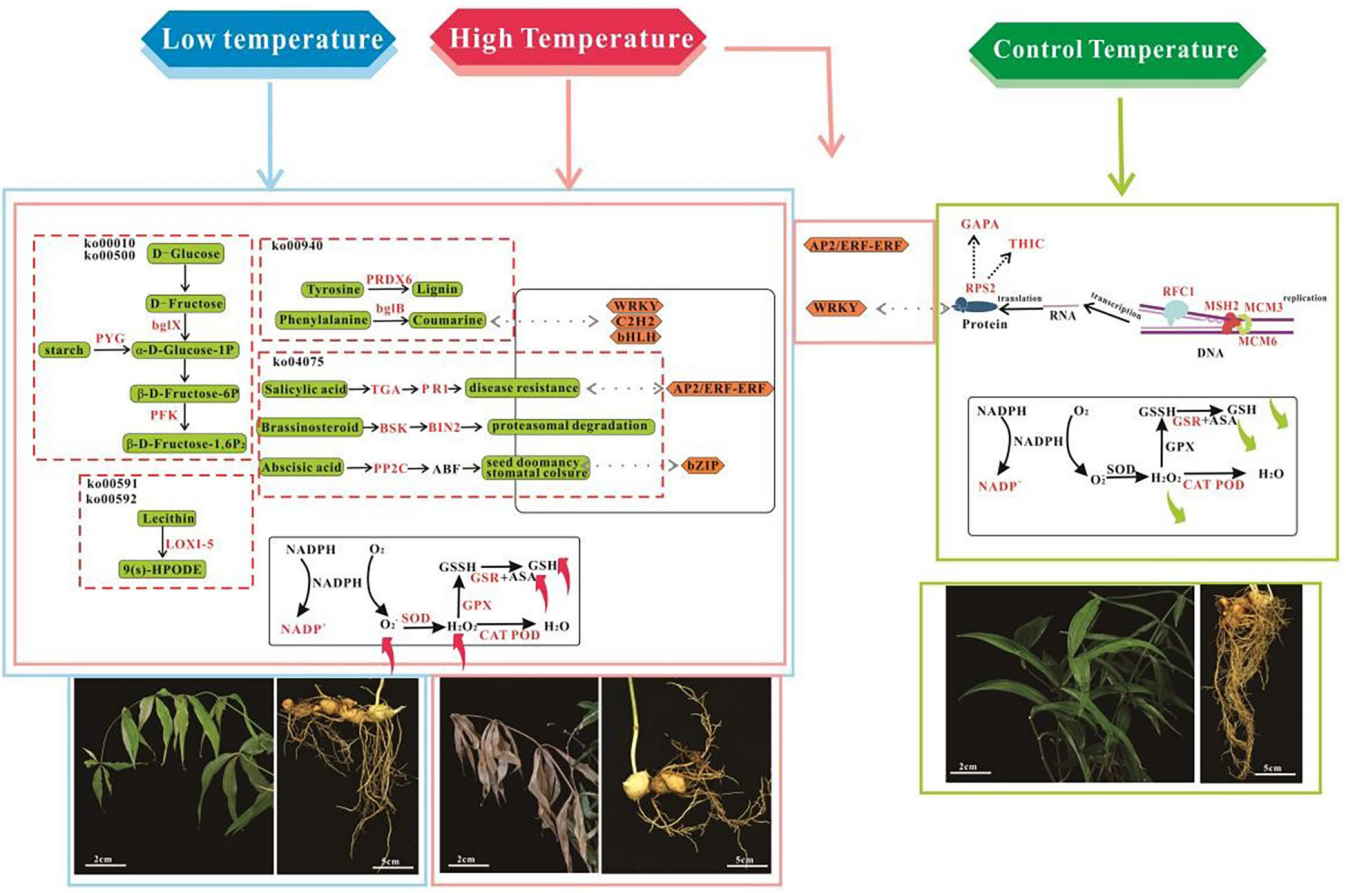
Response mechanisms of *P. kingianum* to temperature stress.

Through integrated analysis of physiological and molecular data from *P. Kingianum* under temperature stress, it was found that the initial response occurs in the leaves (**Figure 9**). Additionally, chlorophyll content decreased with prolonged stress exposure. Conversely, levels of ROS, MDA, and Pro increased, indicating aggravated oxidative damage during the early stages of stress. However, the feedback activation of the antioxidant system—such as increased activity of SOD, CAT, POD, and elevated levels of ASA and GSH—gradually mitigated the damage. This aligns with the stress-induced upregulation mechanisms of SOD/APX observed in white clover and soybean (Liu et al., 2021; Bai et al., 2025). *P. Kingianum* forms complex molecular network to respond to temperature stress by activating key enzyme expression of antioxidant system, activating stress-resistance related pathways and depends on the coordinated regulation of transcription factors. Finally, it drives significant differential expression of stress-responsive genes at transcriptional level. In this study, antioxidant enzymes (SOD, CAT, and POD) and antioxidants (ASA and GSH) in the antioxidant system were selected as physiological response indicators for *P. kingianum* under temperature stress. By combining physiological and transcriptome data, a total of 19 genes encoding key enzymes of the antioxidant system were identified (**Supplementary Table S8**, **Supplementary Figure S3**). Among them, 3 were selected from leaves, 4 from stems, and 12 from tubers. POD catalyzes the decomposition of H_2_O_2_, promotes the synthesis of phenylpropanoid compounds, and thereby enhances the plant’s defense against temperature stress (Liu et al., 2023). Gene downregulation under temperature stress leads to reduced POD synthesis and decreased stress tolerance. In grapes, POD is considered a multifunctional enzyme that can participate in cell wall construction and disease resistance by oxidizing a variety of substrates (Barceló et al., 2003). In stems, the genes encoding peroxidase, TRINITY_DN29145_c0_g1_i7_5, TRINITY_DN30155_c0_g1_i10_12, and TRINITY_DN35306_c0_g1_i10_10, were significantly downregulated under low-temperature (LT), moderate-temperature (MT), and high-temperature (HT) conditions. However, in tubers, a total of 9 genes encoding peroxidase were identified, among them TRINITY_DN10416_c0_g1_i1_8 and TRINITY_DN27733_c1_g1_i2_8 were also significantly up-regulated under three temperature stress conditions, which was consistent with the changes in the enzyme activity of peroxidase. It is believed that these two genes can respond to temperature stress by positively regulating the enzyme activity of peroxidase. The gene encoding catalase, TRINITY_DN29704_c1_g1_i10_2, was significantly upregulated under LT and MT conditions. In contrast, its expression was downregulated under HT conditions, which was consistent with the detection results of catalase. The enzyme activity was highest under low temperature treatment, followed by moderate temperature treatment, and the lowest under high temperature treatment, indicating that gene expression can be activated under low temperature and high temperature conditions, thereby increasing enzyme activity (Bu et al., 2025). In tubers, the gene TRINITY_DN34830_c0_g1_i13_8 encoding glutathione peroxidase was significantly upregulated under all temperature stress (**Supplementary Table S8**). By integrating the physiological data of hydrogen peroxide, the activity of glutathione peroxidase (GPX) is enhanced, increasing its capacity to eliminate hydrogen peroxide. This reduction in hydrogen peroxide levels mitigates the impact of oxidative stress on cells. In leaves, the gene TRINITY DN22948_c1_g4 i1_12 encodes glutathione reductase (GR), whose expression is significantly upregulated under LT, MT, and HT treatments. Glutathione reductase (GR) reduces oxidized glutathione, thereby enhancing the plant’s antioxidant capacity through the regulation of its activity. GPX scavenges ROS directly and GR supports GPX indirectly by maintaining GSH levels to form an antioxidant cycle (Rochette et al., 2022). The antioxidant pathway mediated by GR shares similarities with the detoxification pathway facilitated by glutathione S-transferase (GST) (Vairetti et al., 2021).

Seventeen genes associated with temperature stress response were identified in the resistance-related pathway. In the phenylpropanoid biosynthesis pathway, genes such as those encoding peroxidase TRINITY_DN10416_c0_g1_i1_8, TRINITY_DN27733_c0_g1_i1_8, encoding cinnamyl alcohol dehydrogenase TRINITY_DN20711_c0_g1_i1_4, and encoding β-glucosidase TRINITY_DN33303_c0_g1_i1_10 were upregulated under temperature stress, thereby synergistically synthesizing lignin. This enzyme oxidizes phenolics to cross-link cell walls and collaborates with GPX (TRINITY_DN34830_c0_g1_i13_8) to alleviate lipid peroxidation (**Figure 9**). The lignin synthesis mechanism, mediated by cinnamyl alcohol dehydrogenase (CAD), resembles POD-mediated cell wall remodeling in grapes (Barceló et al., 2003). In the starch and sucrose metabolism pathway, genes such as TRINITY_DN21642_c0_g2_i2_4 (encoding glycogen phosphorylase, PYG) and TRINITY_DN31394_c0_g2_i2_10 (encoding β-glucosidase, bg1X) were upregulated. These enzymes are involved in glycogen breakdown and hydrolysis of β-D-glycosidic bonds, respectively, indicating that carbohydrates in *P. kingianum* are catabolized under temperature stress to increase osmolyte content and enhance osmotic adjustment, thereby improving stress tolerance (Gao et al., 2022). Secondly, to elucidate the synergistic regulatory mechanisms of these stress response pathways, we employed weighted gene co-expression network analysis (WGCNA) to correlate transcriptional modules with physiological phenotypes. In the phenylpropanoid biosynthesis pathway, HT vs CT modules were significantly correlated with ASA and H_2_O_2_. TRINITY_DN34221_c0_g1_i5_9, the core gene of blue module, was the rate-limiting enzyme of phenylpropane metabolism. Its up-regulation under high temperature was significantly and positively correlated with ASA accumulation, indicating that it can scavenge ROS by promoting flavonoid synthesis, which is consistent with the function of Arabidopsis HXK1 in oxidative stress (Vítek et al., 2022). In addition, TRINITY_DN28650_c0_g1_i1_5, another core gene in the core of blue module, is positively correlated with hydrogen peroxide, and has similar function to rice OsPRX114, catalyzing the decomposition of H_2_O_2_. Its up-regulation may reflect the positive feedback regulation of oxidative stress under high temperature (Ma et al., 2025). The turquoise module core gene TRINITY_DN29907_c0_g1_i3_2 is significantly and negatively correlated with ASA accumulation and encodes a coumarate glycosyltransferase functionally similar to Nicotiana tabacum NtTOGT1, enhancing its stability by glycosylation of phenolic compounds (Wang et al., 2012). It can glycosylate phenolic substances, prolong the antioxidant activity cycle, and indirectly reduce the regeneration pressure of ASA (Neugart and Bumke-Vogt, 2021). In starch and sucrose metabolism, the HT vs CT turquoise module is negatively correlated with ASA, Pro, and H_2_O_2_, and its core gene TRINITY_DN31510_c0_g1_i2_9 is homologous to potato StBAM1, which catalyzes the catabolism of starch to generate maltose, and supports osmotic regulation to provide the carbon backbone and energy for ASA and Pro synthesis (Kulakova et al., 2022). However, the current WGCNA analysis primarily focused on leaf tissues. Future studies should extend these analyses to tubers and stems of *P. kingianum* to uncover tissue-specific resistance mechanisms and provide a more comprehensive understanding of its temperature stress response.

At the level of transcriptional regulation, Transcription factors (TFs) such as AP2/ERF may participate in salicylic acid (SA)-induced plant hormone signal transduction and the activation of the phenylpropanoid biosynthesis pathway (Cao et al., 2024; Dutta et al., 2025). In tubers, genes TRINITY_DN24201_c0_g1_i3_6 and TRINITY_DN25872_c0_g1_i1_7 were significantly upregulated under both low and high temperature stress, suggesting their role in enhancing antioxidant capacity through the SA signaling pathway (**Supplementary Table S7**). This activation promotes the synthesis of cinnamyl alcohol dehydrogenase (CAD), encoded by TRINITY_DN20711_c0_g1_i1_4. Meanwhile, WRKY TFs may be involved in abscisic acid (ABA)-induced plant hormone signaling. In leaves, the expression of TRINITY_DN18824_c0_g1_i1_0 was significantly down regulated under temperature stress, potentially suppressing the ABA signaling pathway to avoid excessive defense responses and conserve resources for growth (**Supplementary Figure S2A**). Similar to observations in wheat and rice, ABA enhances the activity of the antioxidant enzyme system and regulates H_2_O_2_ accumulation in response to stress (Yu et al., 2020). TFs such as WRKY and AP2/ERF negatively and positively regulate temperature stress resistance, respectively, while bZIP, bHLH, and C_2_H_2_ TFs positively modulate the thermotolerance of *P. kingianum*. Under high-temperature stress, AP2/ERF TFs and five WRKY genes were significantly highly expressed.

### 4.3 Integrating physiological and molecular insights: implications for sustainable cultivation of *P. kingianum*

This study confirmed from the physiological and molecular biological point of view that the suitable growth temperature range of *P. kingianum* is 10-25 °C. Too high or too low temperature will inhibit the growth of roots and cause the decrease of chlorophyll content, and at the same time trigger stress reactions such as oxidative damage. We identified 19 key genes encoding antioxidant enzymes (POD, CAT, etc.), which are important mediators in the regulation of heat tolerance and can provide genetic targets for molecular marker-assisted breeding or gene editing to improve stress-resistant varieties. In addition, transcription factors (such as AP2/ERF, WRKY, etc.) in SA and ABA signaling pathways are also involved in stress response, which provides a new strategy for molecular breeding or exogenous hormone application to improve plant stress resistance. At present, *P. kingianum* is gradually popularized and planted in many places in Yunnan Province, from Xishuangbanna in tropical areas to Lijiang in alpine areas (Luo et al., 2024). However, there are risks in blindly expanding the planting range. Priority should be given to selecting areas with high annual rainfall and suitable temperature and avoiding unprotected planting in high temperature areas such as Xishuangbanna or high altitude areas such as Lijiang (Meng et al., 2025). In practice, it is suggested to combine temperature threshold with precipitation, altitude, and other environmental factors to implement precise cultivation division (Zhao et al., 2023; Yang et al., 2022). At the same time, a simple early warning system can be established by combining visible stress symptoms such as leaf yellowing and blight with real-time temperature monitoring, so as to take timely regulation measures such as shading and irrigation. The results of this study provide important theoretical and practical basis for planting regionalization, stress resistant variety breeding, and industrial sustainable development of *P. kingianum*.

## 5 Conclusion

In summary, this study delineates the physiological, biochemical, and molecular mechanisms underpinning the temperature stress response in *P. kingianum*. Temperature stress inhibited root growth and reduced photosynthetic efficiency, evidenced by declines in chlorophyll a, b, and total chlorophyll content. Concurrently, it induced the accumulation of ROS; including superoxide anion and H_2_O_2_ and MDA, leading to increased cell membrane permeability. To mitigate oxidative damage, the activities of antioxidant enzymes (SOD, POD, and CAT) and the levels of non-enzymatic antioxidants (ASA and GSH) increased, constituting a coordinated oxidative defense system. At the molecular level, transcriptome analysis identified 17 differentially expressed genes implicated in phenylpropanoid biosynthesis, central carbon metabolism, lipid metabolism, and plant hormone signaling pathways, collectively modulating stress resistance. Furthermore, 19 genes encoding key antioxidant enzymes were characterized: three expressed in leaves, four in stems, and twelve in tubers. These encode enzymes such as POD, CAT, and GR, and their expression patterns correlated with physiological and biochemical changes, collectively regulating adaptive responses to abiotic stress. Transcription factors, including AP2/ERF and WRKY, modulated stress responses via SA and ABA signaling pathways, while bZIP, bHLH, and C_2_H_2_ zinc finger proteins were associated with enhanced stress tolerance (**Figure 9**). These integrated results provide a theoretical foundation for understanding the temperature stress adaptation of *P. kingianum* and support the development of stress-resistant varieties and adaptive cultivation strategies.

## Data Availability

The data presented in this study are publicly available. The data can be found here: https://www.ncbi.nlm.nih.gov/bioproject, accession PRJNA999946.
