# Supplementary material for: Response mechanisms of Polygonatum kingianum to temperature stress: implications for cultivation practices
Source: Front Plant Sci. 2025 Nov 26;16:1645666. doi: 10.3389/fpls.2025.1645666 (PMC12689520; doi:10.3389/fpls.2025.1645666)
Supplement: Supplementary file 1 [file DataSheet1.pdf]

# Supplementary Materials

## 1. Supplementary Table

**Table S1.** qRT-PCR Primer table.

Real-time quantitative primer sequences

| Gene name | Primer Sequence (5'-3') |
|---|---|
| TRINITY_DN10416_c0_g1_i1_8-F | CTTCTCCGACCGCCTCTACT |
| TRINITY_DN10416_c0_g1_i1_8-R | TTGAACAATCCCTTCCACTCC |
| TRINITY_DN10725_c0_g1_i2_12-F | CAATACTGTGATTCGGGAGAT |
| TRINITY_DN10725_c0_g1_i2_12-R | CACCACCAATAATGTAGACCTG |
| TRINITY_DN15731_c0_g1_i2_4-F | AAGGCGGCGGAGCAC |
| TRINITY_DN15731_c0_g1_i2_4--R | AGGTCGGCGGTGTCGTA |
| TRINITY_DN20711_c0_g1_i1_4-F | TAATGCCTTGTTTCTTGGTGAT |
| TRINITY_DN20711_c0_g1_i1_4-R | CTTGAGCAGGTTCCTTCTTTC |
| TRINITY_DN23554_c2_g1_i10_12-F | CAATACTGTGATTCGGGAGAT |
| TRINITY_DN23554_c2_g1_i10_12-R | CACCACCAATAATGTAGACCTG |
| TRINITY_DN27733_c1_g1_i2_8-F | ACTTAGCACCGTTGGACCTC |
| TRINITY_DN27733_c1_g1_i2_8-R | CGCCGTATGCCTTCACTT |
| TRINITY_DN28634_c0_g1_i6_4-F | AGCAGATGATGATGGCAAGTG |
| TRINITY_DN28634_c0_g1_i6_4-R | TGACCGTGTTCGCTGGAGA |
| TRINITY_DN28755_c0_g1_i6_6-F | TCAGCGTGACATTCAAGTGG |
| TRINITY_DN28755_c0_g1_i6_6-R | AAGAGTGAGGCTCGTGAGATAAA |
| TRINITY_DN31554_c0_g2_i11_9-F | GTGGCATAAGGTTGGCATTT |
| TRINITY_DN31554_c0_g2_i11_9-R | GGTGATAGGGAGGTGTTGGAC |
| TRINITY_DN33303_c0_g1_i1_10-F | GCTACGGAGCCTTACATTG |
| TRINITY_DN33303_c0_g1_i1_10-R | GCTGCTGCTTCGTCATCT |
| Actin 7-F | CTCCAGAATCCTTCCAAA R |
| Actin 7-R | GAGAAGAGGGTAGGAGG |

**Table S2.** List of genes related to key metabolic pathway screening.

| Gene id | Organization | Pathway | KEGG gene name | KEGG description | Log2 FC（LT vs CT） | Regulation | Log2 FC (MT vs CT) | Regulation | Log2FC (HT vs CT) | Regulation | Subfigure in Fig. 5 |
|---|---|---|---|---|---|---|---|---|---|---|---|
| TRINITYDN15731_c0_g1_i2_4 | stem | ko00591 | PRDX6 | peroxiredoxin 6, 1-Cys peroxiredoxin [EC:1.11.1.7 1.11.1.15 3.1.1.-] | 4.62 | Up | 5.6 | Up | 3.58 | Up | Fig.5 A |
| TRINITY_DN30155_c0_g1_i10_12 | | ko00940 | E1.11.1.7 | peroxidase [EC:1.11.1.7] | -1.40799 | Down | -1.70327 | Down | -1.09449 | Down | |
| TRINITY_DN23511_c0_g1_i5_12 | | ko00940 | E2.1.1.104 | caffeoyl-CoA O-methyltransferase [EC:2.1.1.104] | -4.86998 | Down | -3.17847 | Down | -5.15948 | Down | |
| TRINITY_DN29145_c0_g1_i7_5 | | ko00940 | E1.11.1.7 | peroxidase [EC:1.11.1.7] | -4.25296 | Down | -3.56477 | Down | -2.74591 | Down | |
| TRINITY_DN35306_c0_g1_i10_10 | | ko00940 | E1.11.1.7 | peroxidase [EC:1.11.1.7] | -3.1171 | Down | -2.38158 | Down | -3.24183 | Down | |
| TRINITY_DN32908_c1_g2_i4_10 | | ko00591 | LOX1_5 | linoleate 9S-lipoxygenase | 7.2 | Up | 5.01 | Up | 7.32 | Up | Fig.5 E |

| | | | | | | | | | | |
|---|---|---|---|---|---|---|---|---|---|---|
| | | | [EC:1.13.11.58] | | | | | | | |
| TRINITY_DN24358_c1_g1_i5_4 | ko00591 | LOX1_5 | linoleate 9S-lipoxygenase [EC:1.13.11.58] | 7.23 | Up | 6.23 | Up | 7.32 | Up | |
| TRINITY_DN28755_c0_g1_i6_6 | ko00591 | LOX1_5 | linoleate 9S-lipoxygenase [EC:1.13.11.58] | 4.19 | Up | 4.38 | Up | 4.45 | Up | |
| TRINITY_DN33920_c0_g1_i14_5 | ko00591 | LOX1_5 | linoleate 9S-lipoxygenase [EC:1.13.11.58] | -2.61747 | Down | -2.27091 | Down | -3.98425 | Down | |
| TRINITY_DN34740_c0_g2_i6_9 | ko00591 | LOX1_5 | linoleate 9S-lipoxygenase [EC:1.13.11.58] | -3.86142 | Down | -2.66968 | Down | -4.68797 | Down | |
| TRINITY_DN33985_c0_g1_i1_11 | ko00591/ko00592 | LOX2S | lipoxygenase [EC:1.13.11.12] | -2.10179 | Down | -3.59207 | Down | -3.44537 | Down | |
| TRINITY_DN26019_c0_g1_i3_12 | ko00591/ko00592 | LOX2S | lipoxygenase [EC:1.13.11.12] | -2.3607 | Down | -3.51988 | Down | -3.44537 | Down | |
| TRINITY_DN29916_c0_g1_i1_2 | ko00591/ko00592 | LOX2S | lipoxygenase [EC:1.13.11.12] | -3.14345 | Down | -4.27705 | Down | -6.24788 | Down | |
| TRINITY_DN29919_c0_g2_i1_1 | ko00592 | E1.3.3.6, ACOX1, ACOX3 | acyl-CoA oxidase [EC:1.3.3.6] | -2.56795 | Down | -2.53486 | Down | -2.10619 | Down | Fig.5F |

| | | | | | | | | | | |
|---|---|---|---|---|---|---|---|---|---|---|
| TRINITY_DN27345_c0_g1_i9_8 | | ko00592 | OPR | 12-oxophytodienoic acid reductase [EC:1.3.1.42] | -3.91758 | Down | -3.03593 | Down | -3.40956 | Down |
| TRINITY_DN31238_c0_g1_i5_6 | | ko00592 | OPR | 12-oxophytodienoic acid reductase [EC:1.3.1.42] | -3.58661 | Down | -4.08523 | Down | -3.37749 | Down |
| TRINITY_DN29976_c0_g2_i1_11 | | ko00592 | AOS | hydroperoxide dehydratase [EC:4.2.1.92] | -1.99465 | Down | -1.15258 | Down | -3.17586 | Down |
| TRINITY_DN30641_c0_g3_i3_5 | | ko00592 | AOS | hydroperoxide dehydratase [EC:4.2.1.92] | -1.61642 | Down | -1.52741 | Down | -2.38343 | Down |
| TRINITY_DN24023_c0_g1_i1_8 | | ko00940 | E1.11.1.7 | peroxidase [EC:1.11.1.7] | -2.20164 | Down | -1.52369 | Down | -2.86711 | Down |
| TRINITY_DN35306_c0_g1_i10_10 | | ko00940 | E1.11.1.7 | peroxidase [EC:1.11.1.7] | -4.07987 | Down | -2.72171 | Down | -3.51978 | Down |
| TRINITY_DN11628_c0_g1_i1_10 | tuber | ko00940 | E1.11.1.7 | peroxidase [EC:1.11.1.7] | -4.89509 | Down | -6.79888 | Down | -6.92388 | Down |
| TRINITY_DN31080_c0_g1_i5_11 | | ko00460/ko00500/ko00940 | bglX | beta-glucosidase [EC:3.2.1.21] | -2.14803 | Down | -2.15274 | Down | -2.65195 | Down |
| TRINITY_DN24473_c1_g1_i11 | | ko00940 | E1.11.1.7 | peroxidase [EC:1.11.1.7] | -4.31434 | Down | -5.16276 | Down | -2.75142 | Down |

| | | | | | | | | | | |
|---|---|---|---|---|---|---|---|---|---|---|
| TRINITY_DN31668_c0_g1_i4_8 | ko00940 | E1.11.1.7 | peroxidase [EC:1.11.1.7] | -1.24646 | Down | -5.16276 | Down | -2.75142 | Down | |
| TRINITY_DN31886_c0_g1_i6_3 | ko00940 | E1.11.1.7 | peroxidase [EC:1.11.1.7] | -1.61555 | Down | -1.40265 | Down | -1.90973 | Down | |
| TRINITY_DN27733_c1_g1_i2_8 | ko00940 | E1.11.1.7 | peroxidase [EC:1.11.1.7] | 2.02 | Up | 2.81 | Up | 3.64 | Up | |
| TRINITY_DN20711_c0_g1_i1_4 | ko00940 | K22395 | cinnamyl-alcohol dehydrogenase [EC:1.1.1.195] | 2.17 | Up | 2.17 | Up | 2.15 | Up | |
| TRINITYDN10416_c0_g1_i1_8 | ko00940 | E1.11.1.7 | peroxidase [EC:1.11.1.7] | 7.34 | Up | 6.58 | Up | 5.8 | Up | |
| TRINITY_DN33303_c0_g1_i1_10 | ko00940 | bglB | beta-glucosidase [EC:3.2.1.21] | 2.39 | Up | 1.57 | Up | 2.43 | Up | |
| TRINITY_DN23554_c2_g1_i10_12 | ko00010/ko00030/ko00051 | pfkA, PFK | 6-phosphofructokinase 1 [EC:2.7.1.11] | 5.02 | Up | 5.83 | Up | 4.9 | Up | |
| TRINITY_DN27880_c0_g2_i10_1 | ko00010/ko00040/ko00561 | AKR1A1, adh | alcohol dehydrogenase (NADP+) [EC:1.1.1.2] | -2.41199 | Down | 1.51575 | Up | 1.300551 | Up | Fig.5 C |
| TRINITY_DN32593_c1_g1_i13_1 | ko00010/ko00020/ko00620 | E4.1.1.49, pckA | phosphoenolpyruvate carboxykinase (ATP) [EC:4.1.1.49] | -4.12496 | Down | -1.5267 | Down | -1.75611 | Down | |

| | | | | | | | | | |
|---|---|---|---|---|---|---|---|---|---|
| TRINITY_DN27469_c0_g4_i1_12 | ko00500/ | pgm | phosphoglucomutase [EC:5.4.2.2] | -8.35287 | Down | -5.69048 | Down | -2.16455 | Down |
| TRINITY_DN34724_c0_g1_i11_10 | ko00010/ko00051/ko00562/ko00710 | TPI, tpiA | triosephosphate isomerase (TIM) [EC:5.3.1.1] | -1.0483 | Down | -1.84409 | Down | -1.18157 | Down |
| TRINITY_DN26341_c0_g2_i4_12 | ko00010/ko00053 | ALDH7A1 | aldehyde dehydrogenase family 7 member A1 [EC:1.2.1.31 1.2.1.8 1.2.1.3] | -8.55909 | Down | -7.13626 | Down | -5.66476 | Down |
| TRINITY_DN34007_c1_g1_i5_10 | ko00010/ko00710 | GAPDH, gapA | glyceraldehyde 3-phosphate dehydrogenase [EC:1.2.1.12] | -8.18271 | Down | -5.79791 | Down | -8.36988 | Down |
| TRINITY_DN30077_c0_g1_i6_12 | ko00010/ko00230/ko00620 | PK, pyk | pyruvate kinase [EC:2.7.1.40] | -1.79007 | Down | -1.18296 | Down | -1.56921 | Down |
| TRINITY_DN34313_c1_g1_i7_5 | ko00010/ko00020/ko00620/ko00710 | E4.1.1.49, pckA | phosphoenolpyruvate carboxykinase (ATP) [EC:4.1.1.49] | -5.06911 | Down | -4.42371 | Down | -5.23712 | Down |
| TRINITY_DN32838_c0_g1_i3_5 | ko00010 | E5.1.3.15 | glucose-6-phosphate 1-epimerase [EC:5.1.3.15] | -1.42774 | Down | -1.63385 | Down | -1.33536 | Down |
| TRINITY_DN25419_c0_g1_i2_5 | ko00010/ko00051/ko00052/ko00500 | HK | hexokinase [EC:2.7.1.1] | -2.42819 | Down | -3.48343 | Down | -2.82012 | Down |

| | | | | | | | | | | |
|---|---|---|---|---|---|---|---|---|---|---|
| TRINITY_DN25532_c0_g1_i1_10 | ko00010 | E5.1.3.15 | glucose-6-phosphate 1-epimerase [EC:5.1.3.15] | -1.7312 | Down | -2.56132 | Down | -2.52753 | Down | |
| TRINITY_DN28634_c0_g1_i6_4 | ko04075 | TGA | transcription factor TGA | 2.12 | Up | 2.37 | Up | 1.61 | Up | |
| TRINITY_DN33532_c0_g6_i2_10 | ko04016/ko04075 | PP2C | protein phosphatase 2C [EC:3.1.3.16] | 4.2 | Up | 4.01 | Up | 4.02 | Up | |
| TRINITY_DN21584_c0_g1_i2_1 | ko04016/ko04075/ko04626 | PR1 | pathogenesis-related protein 1 | 3.27 | Up | 5.06 | Up | 6.48 | Up | |
| TRINITYDN18337_c0_g1_i1_9 | ko04075 | PR1 | pathogenesis-related protein 1 | 1.74 | Up | 2.04 | Up | 1.97 | Up | |
| TRINITYDN31554_c0_g2_i1_19 | ko04075 | BIN2 | protein brassinosteroid insensitive 2 [EC:2.7.11.1] | 1.54 | Up | 1.54 | Up | 1.75 | Up | Fig.5G |
| TRINITY_DN33204_c0_g3_i1_11 | ko04075 | BSK | BR-signaling kinase [EC:2.7.11.1] | 7.04 | Up | 7.78 | Up | 8.25 | Up | |
| TRINITY_DN12668_c0_g1_i1_5 | ko04075 | AUX1, LAX | auxin influx carrier (AUX1 LAX family) | -4.64111 | Down | -3.98699 | Down | -2.81687 | Down | |
| TRINITY_DN30789_c0_g1_i2_1 | ko04075/ko04712 | PIF3 | phytochrome-interacting factor 3 | -2.16799 | Down | -1.18342 | Down | -1.34717 | Down | |
| TRINITY_DN30514_c0_g1_i3_5 | ko04075 | JAZ | jasmonate ZIM domain- | -3.42138 | Down | -1.46679 | Down | -2.0931 | Down | |

| | | | | | | | | | | | |
|---|---|---|---|---|---|---|---|---|---|---|---|
| | | | | containing protein | | | | | | | |
| TRINITY_DN27964_c0_g9_i1_1 | | ko04075 | AUX1, LAX | auxin influx carrier (AUX1 LAX family) | -4.96428 | Down | -5.17981 | Down | -7.0293 | Down | |
| TRINITY_DN28603_c0_g1_i2_1 | | ko04016/ko04075 | PP2C | protein phosphatase 2C [EC:3.1.3.16] | -1.43445 | Down | -1.32697 | Down | -1.89074 | Down | |
| TRINITY_DN25488_c0_g3_i1_1 | | ko04075 | IAA | auxin-responsive protein IAA | -3.59196 | Down | -4.3112 | Down | -5.11507 | Down | |
| TRINITY_DN21624_c0_g2_i2_4 | | ko00500 | PYG, glgP | glycogen phosphorylase [EC:2.4.1.1] | 6.67 | Up | 9.04 | Up | 9.96 | Up | |
| TRINITY_DN31394_c0_g2_i2_10 | | ko00500/ko00940 | bglX | beta-glucosidase [EC:3.2.1.21] | 8.02 | Up | 4.4 | Up | 3.98 | Up | |
| TRINITY_DN24914_c0_g1_i1_2 | | ko00500 | E3.2.1.2 | beta-amylase [EC:3.2.1.2] | 3.371001 | Up | -2.02533 | Down | -1.15295 | Down | |
| TRINITY_DN32509_c0_g1_i1_5 | leaf | ko00500 | ISA, treX | isoamylase [EC:3.2.1.68] | -2.30413 | Down | -6.74762 | Down | -6.19987 | Down | Fig.5 D |
| TRINITY_DN32899_c1_g3_i2_5 | | ko00500 | malQ | 4-alpha-glucanotransferase [EC:2.4.1.25] | -6.38139 | Down | -7.59719 | Down | -4.76119 | Down | |
| TRINITY_DN32357_c0_g2_i2_1 | | ko00500 | malQ | 4-alpha-glucanotransferase [EC:2.4.1.25] | -7.72808 | Down | -9.00173 | Down | -9.03056 | Down | |

| | | | | | | | | | |
|---|---|---|---|---|---|---|---|---|---|
| TRINITY_DN34651_c1_g2_i2_5 | ko00500 | E2.4.1.13 | sucrose synthase [EC:2.4.1.13] | -8.65937 | Down | -8.99458 | Down | -9.02034 | Down |

**Table S3.** Key genes table for key metabolic pathway screening.

| Gene id | Organization | Pathway | KEGG gene name | KEGG description | Log2FC（LT vs CT） | Regulation | Log2FC (MT vs CT) | Regulation | Log2FC (HT vs CT) | Regulation |
|---|---|---|---|---|---|---|---|---|---|---|
| TRINITY_DN21624_c0_g2_i2_4 | leaf | ko00500 | PYG, glgP | glycogen phosphorylase [EC:2.4.1.1] | 6.67 | Up | 9.04 | Up | 9.96 | Up |
| TRINITY_DN31394_c0_g2_i2_10 | | | bglX | beta-glucosidase [EC:3.2.1.21] | 8.02 | Up | 4.40 | Up | 3.98 | Up |
| TRINITY_DN15731_c0_g1_i2_4 | stem | ko00591 | PRDX6 | peroxiredoxin 6, 1-Cys peroxiredoxin [EC:1.11.1.7 1.11.1.15 3.1.1.-] | 4.62 | Up | 5.60 | Up | 3.58 | Up |
| TRINITY_DN24358_c1_g1_i5_4 | | ko00592 | LOX1_5 | linoleate 9S-lipoxygenase [EC:1.13.11.58] | 4.19 | Up | 4.38 | Up | 4.45 | Up |
| TRINITY_DN28755_c0_g1_i6_6 | | | LOX1_5 | linoleate 9S-lipoxygenase [EC:1.13.11.58] | 7.23 | Up | 6.23 | Up | 7.32 | Up |
| TRINITY_DN32908_c1_g2_i4_10 | | | LOX1_5 | linoleate 9S-lipoxygenase [EC:1.13.11.58] | 7.20 | Up | 5.01 | Up | 7.32 | Up |
| TRINITY_DN10416_c0_g1_i1_8 | tuber | ko00940 | E1.11.1.7 | peroxidase [EC:1.11.1.7] | 7.34 | Up | 6.58 | Up | 5.80 | Up |
| TRINITY_DN20711_c0_g1_i1_4 | | | K22395 | cinnamyl-alcohol dehydrogenase [EC:1.1.1.195] | 2.17 | Up | 2.17 | Up | 2.15 | Up |
| TRINITY_DN27733_c1_g1_i2_8 | | | E1.11.1.7 | peroxidase [EC:1.11.1.7] | 2.02 | Up | 2.81 | Up | 3.64 | Up |
| TRINITY_DN33303_c0_g1_i1_10 | | | bglB | beta-glucosidase [EC:3.2.1.21] | 2.39 | Up | 1.57 | Up | 2.43 | Up |
| TRINITY_DN23554_c2_g1_i10_12 | | ko00010 | pfkA, PFK | 6-phosphofructokinase 1 [EC:2.7.1.11] | 5.02 | Up | 5.83 | Up | 4.90 | Up |
| TRINITY_DN18337_c0_g1_i1_9 | | ko04075 | PR1 | pathogenesis-related protein 1 | 1.74 | Up | 2.04 | Up | 1.97 | Up |
| TRINITY_DN21584_c0_g1_i2_1 | | | PR1 | pathogenesis-related protein 1 | 3.27 | Up | 5.06 | Up | 6.48 | Up |

| | | | | | | | | |
|---|---|---|---|---|---|---|---|---|
| TRINITY_DN28634_c0_g1_i6_4 | TGA | transcription factor TGA | 2.12 | Up | 2.37 | Up | 1.61 | Up |
| TRINITY_DN31554_c0_g2_i11_9 | BIN2 | protein brassinosteroid insensitive 2 [EC:2.7.11.1] | 1.54 | Up | 1.54 | Up | 1.75 | Up |
| TRINITY_DN33204_c0_g3_i1_11 | BSK | BR-signaling kinase [EC:2.7.11.1] | 7.04 | Up | 7.78 | Up | 8.25 | Up |
| TRINITY_DN33532_c0_g6_i2_10 | PP2C | protein phosphatase 2C [EC:3.1.3.16] | 4.20 | Up | 4.01 | Up | 4.02 | Up |

**Table S4.** Screening core genes table through weighted gene co-expression network analysis.

| Group | Gene id | KEGG gene name | KEGG description | Pathway | Log2FC | Regulation |
|---|---|---|---|---|---|---|
| HT vs CT (blue） | TRINITY_DN34221_c0_g1_i5_9 | 4CL | 4coumarateCoA ligase [EC:6.2.1.12] | ko00940 | 1.09 | UP |
| | TRINITY_DN16683_c0_g1_i1_9 | CAD | Cinnamyl alcohol dehydrogenase [EC:1.1.1.195] | ko00940 | 4.68 | Up |
| | TRINITY_DN33303_c0_g1_i1_10 | bglB | betaglucosidase [EC:3.2.1.21] | ko00500 | 4.41 | Up |
| HT vs CT (turquoise） | TRINITY_DN29907_c0_g1_i3_2 | TOGT1 | scopoletin glucosyltransferase [EC:2.4.1.128] | ko00940 | 3.08 | Down |
| | TRINITY_DN31510_c0_g1_i2_9 | E3.2.1.2 | betaamylase [EC:3.2.1.2] | ko00500 | 1.35 | Down |
| MT vs CT (blue） | TRINITY_DN31394_c0_g2_i2_10 | bglX | betaglucosidase [EC:3.2.1.21] | ko00500 | 4.40 | Up |
| MT vs CT (turquoise） | TRINITY_DN32509_c0_g1_i1_5 | ISA, treX | isoamylase [EC:3.2.1.68] | ko00500 | 6.74 | Down |
| LT vs CT (blue） | TRINITY_DN28436_c0_g2_i2_5 | MFP2 | enoylCoA hydratase/3hydroxyacyl CoA dehydrogenase [EC:4.2.1.17 1.1.1.35 1.1.1.211] | ko00592 | 1.96 | Up |
| | TRINITY_DN34238_c0_g1_i1_2 | E1.3.3.6, ACOX1, ACOX3 | acylCoA oxidase [EC:1.3.3.6] | ko00592 | 2.29 | Up |
| LT vs CT (turquoise） | TRINITY_DN30857_c0_g2_i1_9 | TOGT1 | scopoletin glucosyltransferase [EC:2.4.1.128] | ko00940 | 3.52 | Down |

**Table S5.** Key genes table were screened by protein-protein interaction (PPI) network analysis.

| Gene id | KEGG gene name | KEGG description | Pathway | Pathway definition |
|---|---|---|---|---|
| TRINITY_DN11775_c0_g1_i1_10 | MCM3 | DNA replication licensing factor MCM3 [EC:3.6.4.12] | ko03030 | DNA replication |
| TRINITY_DN18160_c0_g1_i1_1 | RPS2 | disease resistance protein RPS2 | ko04626 | Plant-pathogen interaction |
| TRINITY_DN20674_c0_g3_i3_1 | MCM6 | DNA replication licensing factor MCM6 [EC:3.6.4.12] | ko03030 | DNA replication |
| TRINITY_DN24695_c1_g1_i3_1 | RPS2 | disease resistance protein RPS2 | ko04626 | Plant-pathogen interaction |
| TRINITY_DN26542_c0_g1_i1_9 | MSH2 | DNA mismatch repair protein MSH2 | ko03430 | Mismatch repair |
| TRINITY_DN27526_c0_g1_i1_10 | RPS2 | disease resistance protein RPS2 | ko04626 | Plant-pathogen interaction |
| TRINITY_DN30672_c0_g1_i6_10 | MCM6 | DNA replication licensing factor MCM6 [EC:3.6.4.12] | ko03030 | DNA replication |
| TRINITY_DN32028_c0_g3_i1_7 | RFC1 | replication factor C subunit 1 | ko03030+ko03420+ko03430 | DNA replication+Nucleotide excision repair+Mismatch repair |
| TRINITY_DN32073_c0_g1_i5_8 | THIC | phosphomethylpyrimidine synthase [EC:4.1.99.17] | ko00730 | Thiamine metabolism |
| TRINITY_DN34007_c1_g1_i5_10 | GAPA | glyceraldehyde 3-phosphate dehydrogenase [EC:1.2.1.12] | ko00010+ko00710 | Glycolysis +Gluconeogenesis+Carbon fixation in photosynthetic organisms |
| TRINITY_DN40511_c0_g1_i1_10 | MCM3 | DNA replication licensing factor MCM3 [EC:3.6.4.12] | ko03030 | DNA replication |

**Table S6.** Relative gene expression levels of key genes table screened by PPI network analysis（$Log_2$Fold Change）.

| Gene id | Log2FC (LT-K vs CT-K) | Regulation | Log2FC (MT-K vs CT-K) | Regulation | Log2FC (HT-K vs CT-K) | Regulation |
|---|---|---|---|---|---|---|
| TRINITY_DN11775_c0_g1_i1_10 | -3.06 | Down | -3.14 | Down | -2.62 | Down |
| TRINITY_DN18160_c0_g1_i1_1 | -2.77 | Down | -2.32 | Down | -1.94 | Down |
| TRINITY_DN20674_c0_g3_i3_1 | -3.38 | Down | -4.51 | Down | -5.30 | Down |
| TRINITY_DN24695_c1_g1_i3_1 | -3.41 | Down | -4.45 | Down | -4.04 | Down |
| TRINITY_DN26542_c0_g1_i1_9 | -1.97 | Down | -2.02 | Down | -1.15 | Down |
| TRINITY_DN27526_c0_g1_i1_10 | -2.16 | Down | -2.84 | Down | -1.71 | Down |
| TRINITY_DN30672_c0_g1_i6_10 | -3.15 | Down | -3.50 | Down | -1.99 | Down |
| TRINITY_DN32028_c0_g3_i1_7 | -7.00 | Down | -7.05 | Down | -4.02 | Down |
| TRINITY_DN32073_c0_g1_i5_8 | -1.30 | Down | -1.69 | Down | -1.69 | Down |
| TRINITY_DN34007_c1_g1_i5_10 | -8.18 | Down | -5.80 | Down | -8.37 | Down |
| TRINITY_DN40511_c0_g1_i1_10 | -2.41 | Down | -4.38 | Down | -2.74 | Down |

**Table S7.** Key transcription factors related to key genes table.

| Gene id | Family | Organization | Log2FC（LT vs CT） | Regulation | Log2FC（MT vs CT） | Regulation | Log2FC（HT vs CT） | Regulation |
|---|---|---|---|---|---|---|---|---|
| TRINITY_DN18824_c0_g1_i1_10 | WRKY | leaf | -4.96 | Down | -3.82 | Down | -4.84 | Down |
| TRINITY_DN21936_c0_g1_i1_6 | C2H2 | stem | 1.74 | Up | 2.20 | Up | 2.19 | Up |
| TRINITY_DN24137_c0_g1_i5_12 | bZIP | tuber | 1.92 | Up | 2.18 | Up | 2.92 | Up |
| TRINITY_DN24201_c0_g1_i3_6 | AP2/ERF-ERF | | 1.44 | Up | 1.23 | Up | 1.11 | Up |
| TRINITY_DN25093_c0_g1_i1_8 | AP2/ERF-ERF | | -3.51 | Down | 1.52 | Up | 1.77 | Up |
| TRINITY_DN25872_c0_g1_i1_7 | AP2/ERF-ERF | | 1.83 | Up | 2.37 | Up | 1.46 | Up |
| TRINITY_DN25873_c0_g1_i5_4 | WRKY | | -3.69 | Down | 1.03 | Up | 2.87 | Up |
| TRINITY_DN26115_c3_g1_i7_12 | C2H2 | | 8.01 | Up | 4.62 | Up | 8.39 | Up |
| TRINITY_DN26484_c0_g1_i6_12 | WRKY | | -2.66 | Down | 1.34 | Up | 1.22 | Up |
| TRINITY_DN27408_c0_g1_i1_12 | C2H2 | | 2.43 | Up | 8.09 | Up | 3.31 | Up |
| TRINITY_DN28088_c1_g2_i2_6 | bHLH | | 1.05 | Up | 2.24 | Up | 1.72 | Up |
| TRINITY_DN28634_c0_g1_i6_4 | bZIP | | 2.12 | Up | 1.70 | Up | 1.61 | Up |
| TRINITY_DN28810_c0_g1_i17_4 | bHLH | | 1.58 | Up | 1.44 | Up | 2.55 | Up |
| TRINITY_DN30161_c0_g1_i5_1 | WRKY | | -4.38 | Down | 1.56 | Up | 1.78 | Up |

| | | | | | | | |
|---|---|---|---|---|---|---|---|
| TRINITY_DN30731_c1_g1_i1_12 | WRKY | -1.28 | Down | 2.03 | Up | 1.38 | Up |

**Table S8.** Key genes table in physio-molecular regulatory network screening.

| Gene id | KEGG gene name | KEGG description | Pathway | Pathway definition |
|---|---|---|---|---|
| TRINITY_DN24867_c0_g1_i12_5 | AKR1A1, adh | alcohol dehydrogenase (NADP+) [EC:1.1.1.] | ko00010+ko00040+ko00561 | Glycolysis + Gluconeogenesis+Pentose and glucuronate interconversions+Glycerolipid metabolism |
| TRINITY_DN32048_c0_g1_i7_7 | AKR1A1, adh | alcohol dehydrogenase (NADP+) [EC:1.1.1.] | ko00010+ko00040+ko00561 | Glycolysis + Gluconeogenesis+Pentose and glucuronate interconversions+Glycerolipid metabolism |
| TRINITY_DN33988_c0_g1_i6_5 | E1.1.1.40, maeB | malate dehydrogenase (oxaloacetate-decarboxylating) (NADP+) [EC:1.1.1.40] | ko00620+ko00710 | Pyruvate metabolism+Carbon fixation in photosynthetic organisms |
| TRINITY_DN10416_c0_g1_i1_8 | E1.11.1.7 | peroxidase [EC:1.11.1.7] | ko00940 | Phenylpropanoid biosynthesis |

| | | | | |
|---|---|---|---|---|
| TRINITY_DN11628_c0_g1_i1_10 | E1.11.1.7 | peroxidase [EC:1.11.1.7] | ko00940 | Phenylpropanoid biosynthesis |
| TRINITY_DN21709_c0_g1_i1_1 | E1.11.1.7 | peroxidase [EC:1.11.1.7] | ko00940 | Phenylpropanoid biosynthesis |
| TRINITY_DN24023_c0_g1_i1_8 | E1.11.1.7 | peroxidase [EC:1.11.1.7] | ko00940 | Phenylpropanoid biosynthesis |
| TRINITY_DN24473_c1_g1_i11_1 | E1.11.1.7 | peroxidase [EC:1.11.1.7] | ko00940 | Phenylpropanoid biosynthesis |
| TRINITY_DN26912_c1_g2_i1_4 | E1.11.1.7 | peroxidase [EC:1.11.1.7] | ko00940 | Phenylpropanoid biosynthesis |
| TRINITY_DN27733_c1_g1_i2_8 | E1.11.1.7 | peroxidase [EC:1.11.1.7] | ko00940 | Phenylpropanoid biosynthesis |
| TRINITY_DN29145_c0_g1_i7_5 | E1.11.1.7 | peroxidase [EC:1.11.1.7] | ko00940 | Phenylpropanoid biosynthesis |
| TRINITY_DN30155_c0_g1_i10_12 | E1.11.1.7 | peroxidase [EC:1.11.1.7] | ko00940 | Phenylpropanoid biosynthesis |
| TRINITY_DN31668_c0_g1_i4_8 | E1.11.1.7 | peroxidase [EC:1.11.1.7] | ko00940 | Phenylpropanoid biosynthesis |
| TRINITY_DN31886_c0_g1_i6_3 | E1.11.1.7 | peroxidase [EC:1.11.1.7] | ko00940 | Phenylpropanoid biosynthesis |

| | | | | |
|---|---|---|---|---|
| TRINITY_DN35306_c0_g1_i10_10 | E1.11.1.7 | peroxidase [EC:1.11.1.7] | ko00940 | Phenylpropanoid biosynthesis |
| TRINITY_DN28114_c0_g1_i1_10 | gpx, btuE, bsaA | glutathione peroxidase [EC:1.11.1.9] | ko00480+ko00590 | Glutathione metabolism+Arachidonic acid metabolism |
| TRINITY_DN34830_c0_g1_i13_8 | gpx, btuE, bsaA | glutathione peroxidase [EC:1.11.1.9] | ko00480+ko00590 | Glutathione metabolism+Arachidonic acid metabolism |
| TRINITY_DN22948_c1_g4_i1_12 | GSR, gor | glutathione reductase (NADPH) [EC:1.8.1.7] | ko00480 | Glutathione metabolism |
| TRINITY_DN29704_c1_g1_i10_2 | katE, CAT, catB, srpA | catalase [EC:1.11.1.6] | ko00380+ko00630+ko04016+ko04146 | Tryptophan metabolism+Glyoxylate and dicarboxylate metabolism+MAPK signaling pathway - plant+Peroxisome |

# 1. Supplementary Figure

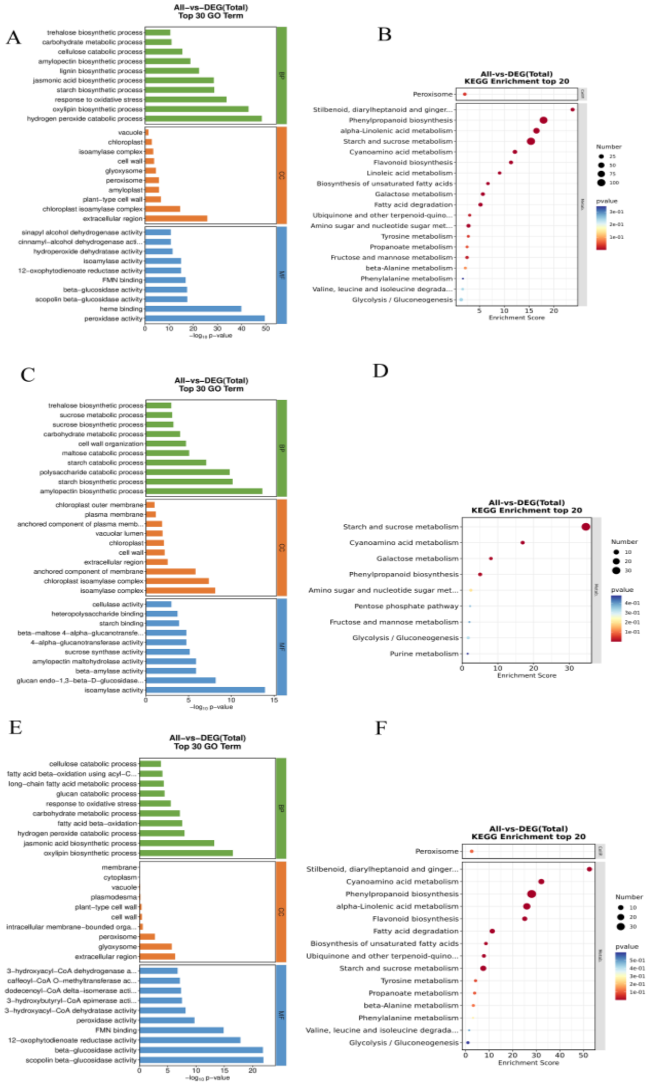

**Figure S1**. In which (A, C, E) represent the GO enrichment analysis for the three comparison groups HT vs CT, MT vs CT, and LT vs CT; (B, D, F) represent the KEGG enrichment analysis for the specific modules related to the weighted co-expression network analysis. The vertical axis of the GO enrichment analysis represents the Top 30 GO enrichment terms, and the horizontal axis represents the -log10(p-value); the vertical axis of the KEGG enrichment analysis represents the Top 20 KEGG enrichment terms, and the horizontal axis represents the KEGG enrichment score.

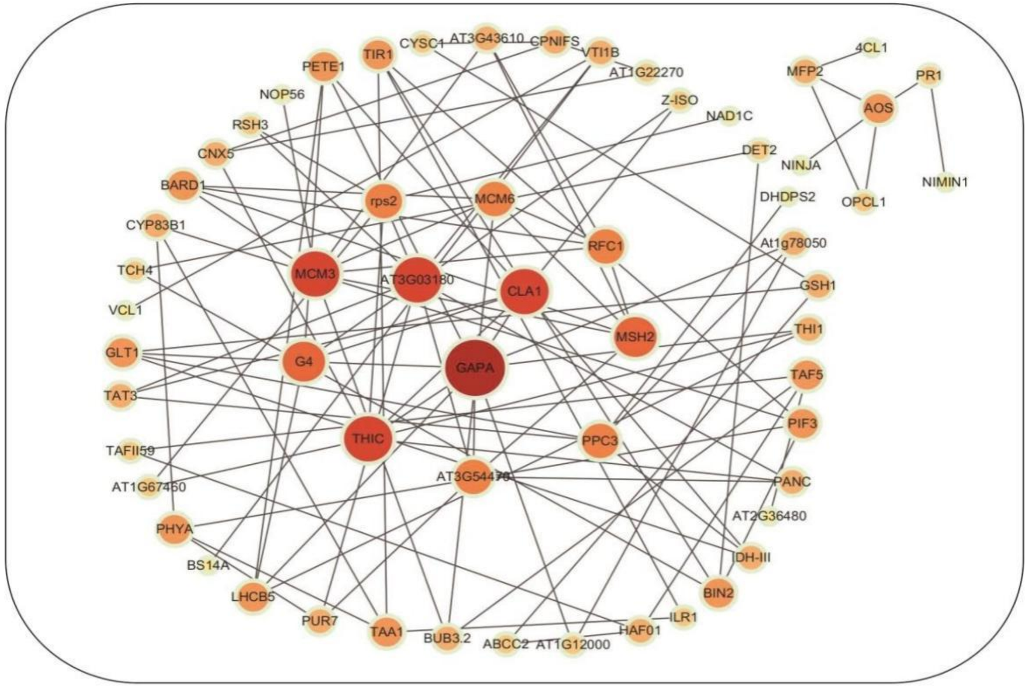


**Figure S2.** Protein–protein interaction (PPI) networks of the DEGs in tubers of *P. kingianum* after exposure to temperature stress were constructed based on the STRING database and Cytoscape software.

Notes: Each node represents a protein, and edges between nodes denote protein-protein interactions. Node size and color (graded from yellow to orange to red) scale with degree centrality, where larger size and more intense red coloration indicate higher connectivity within the network.

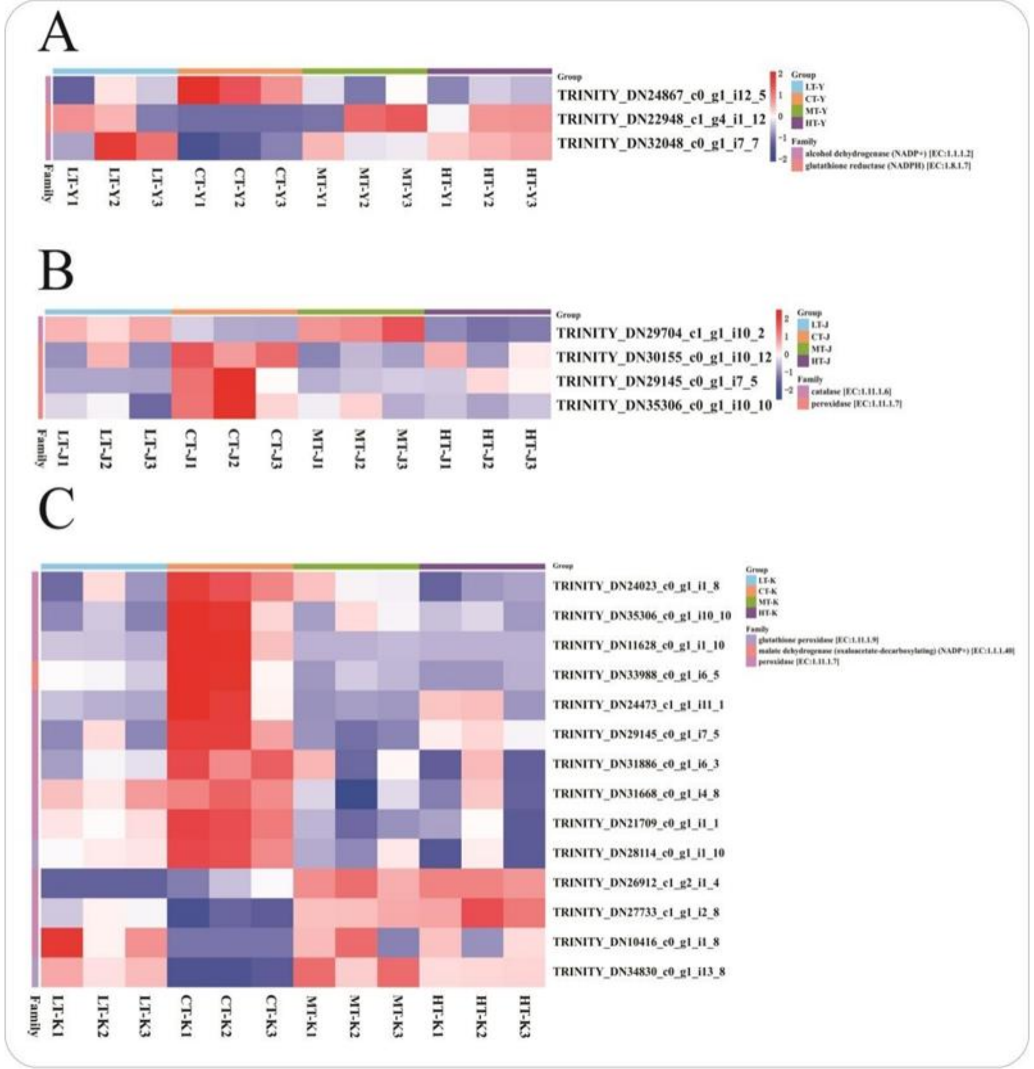


**Figure S3**. Heatmap showing the expression profiles of key genes screened based on integrated physiological and molecular data. (A) Key genes screened in leaves; (B) key genes screened in stems; (C) key genes screened in tubers.
